# Investment case for small and sick newborn care in Tanzania: systematic analyses

**DOI:** 10.1186/s12887-023-04414-2

**Published:** 2023-12-14

**Authors:** Rosemary Kamuyu, Alice Tarus, Felix Bundala, Georgina Msemo, Donat Shamba, Catherine Paul, Robert Tillya, Sarah Murless-Collins, Maria Oden, Rebecca Richards-Kortum, Timothy Powell-Jackson, Meghan Bruce Kumar, Nahya Salim, Joy E Lawn

**Affiliations:** 1 Maternal, Adolescent, Reproductive & Child Health (MARCH) Centre, London School of Hygiene & Tropical Medicine, London, UK; 2 Newborn, Child and Adolescent Health Section, Division of Reproductive, Maternal and Child Health, Ministry of Health, Dar es Salaam, United Republic of Tanzania; 3 Global Financing Facility, the World Bank Group, Washington D.C., USA; 4 Department of Health Systems, Impact Evaluation and Policy, Ifakara Health Institute, Dar es Salaam, United Republic of Tanzania; 5 Rice360 Institute for Global Health Technologies, Rice University, Texas, USA; 6 Kenya Medical Research Institute–Wellcome Trust Research Program, Nairobi, Kenya; 7 Muhimbili University of Health and Allied Sciences, Dar es Salaam, Tanzania

**Keywords:** Newborn, Neonatal, Low- and middle-income countries, Hospital care, Investment case, Return on investment

## Abstract

**Background:**

Small and sick newborn care (SSNC) is critical for national neonatal mortality reduction targets by 2030. Investment cases could inform implementation planning and enable coordinated resource mobilisation. We outline development of an investment case for Tanzania to estimate additional financing for scaling up SSNC to 80% of districts as part of health sector strategies to meet the country’s targets.

**Methods:**

We followed five steps: (1) reviewed national targets, policies and guidelines; (2) modelled potential health benefits by increased coverage of SSNC using the Lives Saved Tool; (3) estimated setup and running costs using the Neonatal Device Planning and Costing Tool, applying two scenarios: (A) all new neonatal units and devices with optimal staffing, and (B) half new and half modifying, upgrading, or adding resources to existing neonatal units; (4) calculated budget impact and return on investment (ROI) and (5) identified potential financing opportunities.

**Results:**

Neonatal mortality rate was forecast to fall from 20 to 13 per 1000 live births with scale-up of SSNC, superseding the government 2025 target of 15, and close to the 2030 Sustainable Development Goal 3.2 target of <12. At 85% endline coverage, estimated cumulative lives saved were 36,600 by 2025 and 80,000 by 2030. Total incremental costs were estimated at US$166 million for scenario A (US$112 million set up and US$54 million for running costs) and US$90 million for scenario B (US$65 million setup and US$25 million for running costs). Setup costs were driven by infrastructure (83%) and running costs by human resources (60%). Cost per capita was US$0.93 and the ROI is estimated to be between US$8–12 for every dollar invested.

**Conclusions:**

ROI for SSNC is higher compared to other health investments, noting many deaths averted followed by full lifespan. This is conservative since disability averted is not included. Budget impact analysis estimated a required 2.3% increase in total government health expenditure per capita from US$40.62 in 2020, which is considered affordable, and the government has already allocated additional funding. Our proposed five-step SSNC investment case has potential for other countries wanting to accelerate progress.

**Supplementary Information:**

The online version contains supplementary material available at 10.1186/s12887-023-04414-2.

## Key findings

**Table:** 

**WHAT WAS KNOWN?**
• With less than a decade to the Sustainable Development Goals (SDGs) deadline, 63 countries, the majority of which are low- and middle-income countries (LMIC), are off track to achieving SDG target 3.2 of reducing newborn deaths to 12 or less per 1000 live births. • Despite newborn deaths contributing to nearly half of all ‘under 5’ deaths globally, domestic and external investment in newborn care is still low. • From official development assistance data with donor analyses from 2002 to 2019, of the US$142M support for Reproductive, Maternal, Newborn and Child Health interventions, only <US$1M was targeted for newborn-specific interventions. • We found no published national investment cases for small and sick newborn care (SSNC) to inform resource allocation for SSNC, especially for African countries which have the biggest survival and investment gaps. • To address this need, we developed an investment case proposing a five-step process based on analyses and learnings from Tanzania, providing evidence-based analyses to inform cost drivers and return on investment for SSNC scale-up
**WHAT WAS DONE THAT IS NEW?**
We developed a five-step framework by adapting guidance from the Global Financing Facility (GFF) investment case framework for Low Middle-Income Countries on how to develop a sound investment case: Step 1: Policy imperatives Step 2: Impact modelling – Using the Lives Saved Tool widely used to estimate health gains in RMNCH interventions. Step 3: Costing – Micro-costing approach using Activity Based Costing Method Step 4: Return on investment (ROI) Step 5: Financing, implementation, resilience, and communication • The five-step process was refined with inputs from the Tanzanian government, multi-disciplinary stakeholders, government guidelines and national standards (e.g., costed floor plans). • Widely available approaches and tools are sufficient to gather required data (e.g., impact estimation tools such as LiST, costing approaches such as Activity Based Costing). • Completion of these analyses for Tanzania was achievable in six months.
**WHAT WAS FOUND?**
• Step 1: Policy imperative- Tanzania’s has committed through its One Plan III 2021/22-2025/26 to reduce the Neonatal Mortality Rate (NMR) to 15 per 1000 live births by 2025 and to 12 or less by 2030. • Step 2: Impact modelling-Tanzania is projected to meet the One Plan III NMR target by 2025 and slightly miss the SDG target by 2030, unless a combined package of care including obstetric and newborn care is implemented. • Step 3: Costing (Set up and running costs) - Infrastructure is the main cost driver but once amortized it is affordable. Human resources, especially nurses’ salaries, drive the running costs. • Step 4: ROI – Cost per death averted was estimated between US$4,297 and US$5,142 by 2030 considering sensitivity analysis of the running costs. For every US$1 invested there is a potential return of between US$8 and US$12 making it a worthwhile investment. • Step 5: Financing, implementation, resilience, and communication – Government and implementing partners’ commitment is key to attain effective coverage.
**WHAT NEXT?**
• Investing in SSNC in Tanzania has the potential for high impact and high return on investment. The budget impact is affordable once the set-up costs are amortized, and the ROI is high. However, delaying investment may result in not meeting the SDG targets by 2030. • Implementation will require a major focus on human resources, especially in rural areas. • The investment case returns are focused on mortality, resulting in an underestimation but there is limited long-term cohort data on neonatal outcomes in African contexts. • Economic research priorities include more formal cost-effectiveness analyses on primary data and method to better track domestic financing for maternal, neonatal, and childcare programmes.

## Background

Investment in small and sick newborn care (SSNC) is needed to achieve the Sustainable Development Goal (SDG) 3.2 target of reducing preventable neonatal deaths to <12 per 1000 live births by 2030 [1, 2]. Globally, 2.3 million newborns die during their first 28 days after birth, accounting for half of the deaths amongst children under 5 years of age [1, 2]. Approximately 98% of these deaths occur in low- and middle-income countries (LMIC) [2], with the leading causes being preterm birth, neonatal infections, and intrapartum injury [1, 3, 4]. Despite the burden of newborn mortality, investment in cost-effective, evidenced-based newborn care interventions is lacking or limited. In 2019, there was US$15.9 billion in official development assistance for reproductive, maternal, newborn, and child health (RMNCH), yet only 10% of official development assistance mentions newborns, and less than 1% includes interventions for neonatal care [5]. As the deadline for the 2030 SDGs approaches, 63 LMIC are off-track to achieve target 3.2 [1]. Political commitment and effective resource mobilisation techniques are urgently required to accelerate progress [6].

The *Every Newborn* Action Plan (ENAP), launched in 2014 was accompanied by a WHO resolution endorsed by all member states, providing a roadmap of strategic actions for ending preventable newborn mortality and stillbirths [4]. ENAP coverage targets call for countries to ensure 80% of districts have at least one level-2 inpatient unit to care for small and sick newborns by 2025, with respiratory support including provision of continuous positive airway pressure (CPAP) (simply referred to as level-2 care in this paper) [5, 7]. Standard of care at this level includes thermal care, kangaroo mother care (KMC) for all stable neonates weighing <2000g, assisted feeding and intravenous fluids, safe administration of oxygen, neonatal sepsis management with injected antibiotics, management of neonatal jaundice with phototherapy, management of neonatal encephalopathy, detection of congenital abnormalities and referral or management of birth defects. Level-2 SSNC also encompasses management of preterm respiratory distress with CPAP, follow-up of at-risk newborns and exchange transfusion [4, 8].

Currently, 106 country governments are committed to reaching newborn survival targets and scaling up SSNC, but there are gaps in implementation, especially in resource allocation [9], noting strained health budgets following the COVID-19 pandemic. However, despite recent focus on neonatal care in hospitals compared to obstetric care, there are major investment gaps for SSNC which require the right space, infrastructure, more skilled health workers especially neonatal nurses, adequate devices, drugs and consumables, and individual-level data to measure and improve quality of care [2, 3, 9, 10]. Notably, across sub–Saharan Africa, many hospitals have no neonatal unit or have a small room with limited devices and staff [10].

With various competing health budget demands, and scarce funding for newborn care, it is essential to examine whether the scale-up of care services for small and sick newborns represents a smart investment for countries. To deliver on political commitments, health planners must identify which interventions to invest in, and at what coverage levels, as well as assess costs and economic returns on these investments. Governments want to prioritise high-return investments, but inadequate data and tools for cost and benefit estimation can hinder evidence-based decision-making for resource allocation [7, 8]. Addressing this gap will provide data for more ambitious investment to get countries back on track towards achieving newborn targets.

### Aim and objectives

This paper is part of a supplement reporting findings and learnings from NEST360, an alliance of partners, including four African governments (Kenya , Malawi, Nigeria and Tanzania), working to reduce neonatal inpatient deaths by improving level-2 newborn care in hospitals. In this paper, we aim to describe the development and results of an investment case to support country commitments to meet the ENAP and SDG targets by scaling level-2 SSNC. The paper addresses two objectives:

- ** 1. **Develop a generic investment case template to estimate the incremental costs, and health and economic benefits of national SSNC scale-up.
- **2.** Apply the template in Tanzania for SSNC scale-up of at least 80% of districts having one unit offering level-2 care (plus CPAP) to meet ENAP target 4. In Tanzania, this translates to 146 District Hospitals and 25 Regional Referral Hospitals.

## Methods

### Overview

**Fig. 1:**
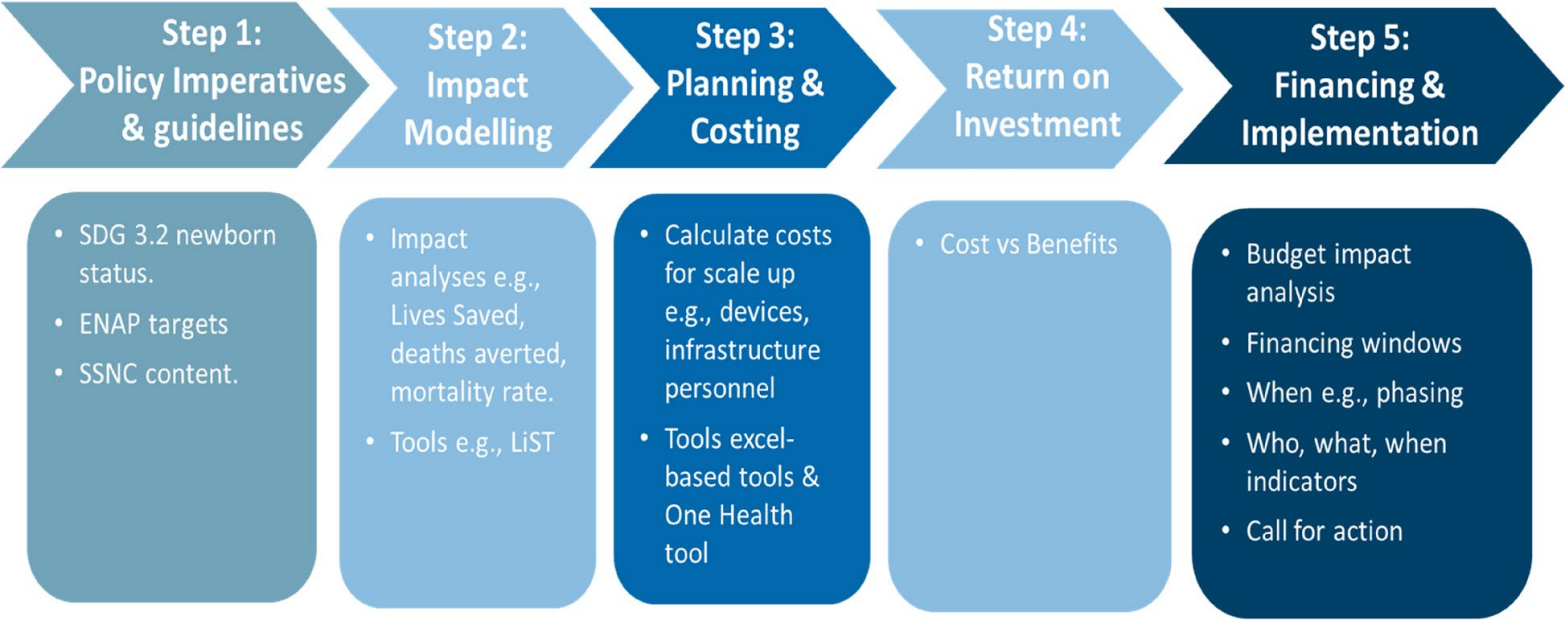
Five-step investment case framework. Abbreviations: SDG; Sustainable Development Goal, ENAP; Every Newborn Action Plan, SSNC; Small and Sick newborn care LiST; Lives Saved Tool. *Adapted from World Bank Global Financing Facility Investment case framework: * [11]

To develop an analytical framework of the SSNC investment case, we adapted a five-step approach from the Global Financing Facility (GFF) investment case framework guidance for LMIC (Fig. 1) [11]. We then applied this five-step framework to develop an investment case for national SSNC scale-up in Tanzania with leadership from the Tanzania Ministry of Health and inputs from a multidisciplinary team of researchers, neonatologists, biomedical experts, clinicians, health economists, health management information systems and quality improvement experts.

### Using the five-step framework to develop an investment case for national SSNC scale-up in Tanzania

#### Step 1: Country policy imperatives and guidelines

We reviewed Tanzanian documentary resources to deduce requirements to deliver level-2 SSNC in the country [12–15]. Inputs included policy documents and guidelines, such as the national One Plan III [12], existing newborn-specific guidelines, and novel national costed newborn unit floor plans for district and regional facilities (Additional file 1), plus guidelines for medicines and devices as well as human resources for health. Tanzania's commitments to improving neonatal survival includes improving intrapartum care and resuscitation, and scale-up of quality Neonatal Care Units with management of sepsis and prematurity, including KMC [16]. Linked systems changes involve Health Management Information Systems data to capture neonatal indicators, neonatal audit, and introduction of competence-based training on newborn health [16].

#### Step 2: Health impact modelling using the Lives Saved Tool (LiST)

We estimated potential health benefits of SSNC scale-up across mainland Tanzania and Zanzibar using LiST. LiST allows users to model potential gains from accelerating coverage of high-impact interventions for RMNCH and is widely used to estimate health gains and inform national plans [17, 18]. LiST has pre-populated data inputs including demographic data and country-specific health status metrics based on the most recent United Nations (UN) estimates and demographic surveys. UN estimates do not separate Mainland Tanzania and Zanzibar; hence, combined estimates were available, precluding ability to calculate separate health benefits. The lives saved estimates are generated by changing coverage levels for evidence-based interventions which have set effectiveness levels for causes-specific mortality impact based on published reviews. When multiple interventions target the same cause of death, the impact is calculated sequentially to avoid double counting the number of prevented deaths [17–19].

The high-impact SSNC interventions included in LiST are based on The Lancet series (Table 1, see further details of the LIST SSNC interventions in Additional file 2). We used the LiST prepopulated number of annual births and neonatal deaths and cause-specific mortality. Based on Tanzania’s annual number of newborn births (i.e., 2.15 million) [16], we estimated that 10% of live births would require SSNC. We applied an average admission of seven days, estimated using NEST360 alliance data in Tanzania [12, 15]. We applied an endline coverage estimate of 85% for all interventions by 2025 and 2030 starting with a baseline (2021) coverage of 10% of all interventions (Table 1). Two scale-up time periods were used for analyses, 2021–2030 for the SDG and 2021–2025 for the current national target in Tanzania.

**Table 1:** Interventions for modelling impact of small and sick newborn care scale-up, using the Lives Saved tool (LiST)

**Interventions included in Small and sick newborn care package**	**Cause of death**	**Intervention Effectiveness**^**a**^** %**	**Baseline Coverage (2021) %**	**Target Coverage 2021 to 2025 and 2021 to 2030 (%)**
Kangaroo Mother Care	Preterm birth direct complications	51 (Note: 47% affected fraction)	10	85
Care for prematurity ((including CPAP/respiratory)		90	10	
Injectable antibiotics for neonatal sepsis	Neonatal infections	90	10	85
Supportive care for neonatal sepsis		90	10	
Supportive care for intrapartum complications	Intrapartum complications (previously called “birth asphyxia”)	10	10	85
Supportive care for congenital conditions	Congenital conditions	10	10	85

**Abbreviations**: *CPAP* Continuous positive airway pressure,

^a^Effect estimated from [2].

#### Step 3: Planning and costing

We employed a normative micro-costing approach using the Activity Based Costing (ABC) approach to estimate incremental costs from a health provider perspective [20, 21]. A novel, ABC Neonatal Device Planning and Costing Tool [22] was used to estimate some of the setup costs related to neonatal furniture and devices. All costs were expressed using 2022 US dollars (1 US$=2,339 Tanzanian shillings (TZS)). Inflation was not incorporated because reporting costs in US dollars help smooth inflationary spikes. Capital assets were annualised using a discount rate of 3% over their useful years; infrastructure for 20 years, and ward furniture and neonatal devices for five years.

Incremental cost estimates included setup and running costs required to scale SSNC to 146 District Hospital and 25 Regional Referral Hospitals (total 171 hospitals) in Mainland Tanzania which would enable reaching the targeted 85% coverage of interventions. Costing was done for Mainland Tanzania only, due to differing governance and budgeting processes compared to Zanzibar. Setup costs included infrastructure, ward furniture and fixtures, and neonatal devices. Running costs included human resources, medical supplies and device consumables, neonatal medicines, maintenance, and data and quality improvement systems (Additional file 3). Most of the cost estimates used were provided by the government and reflected actual costs they had incurred, resulting in minimal uncertainty.

#### Scale-up costing scenarios

Two scenarios were simulated both with a baseline year of 2021. Scenario A would be the ‘best case’; with all new infrastructure and devices, fully adhering to optimal national guidelines such as nurse to baby ratios. Scenario B assumed a ‘halfway case’; with half of buildings and devices new and the remainder renovated, and halfway to the optimal HR ratios.

*Scenario A* considered the incremental setup costs of establishing completely new SSNC units in 171 hospitals, including the acquisition of new ward furniture, fixtures, and neonatal devices needed for level-2 and partial level-3 SSNC care at District Hospital and Regional Referral Hospitals (Additional file 4) [23]. We used existing government costed floor plans to estimate infrastructure costs. Running costs were based on operationalising each newborn unit, adhering to all government standards for quality care provision. Table 2 outlines how each input for both set up and running costs were estimated.

**Table 2:** Summary and data sources for small and sick newborn care scale-up per scenario

**Cost category**	**Type of cost**	**List and incremental quantities estimate**	**Incremental unit costs estimate**	**Costing assumption scenario A**	**Costing assumption scenario B**
Setup Costs	Infrastructure	Government newborn floor plans: -DH: 40-bed unit capacity. -RRH: 80-bed capacity.	Costed approved newborn unit floor plans: -DH: approximately US$ 358,000 -RRH: approximately US$ 1.6 million	Building all new (171) health facilities.	Building half new structures (86) and half renovating. The renovation cost was assumed to be 20% cost of a new structure.
	Ward Furniture & Fixtures	-DH: List of 18 items. -RRH: List of 18 items.	Median unit cost from local supplier’s price list.	Purchase all items new for all health facilities.	Acquire half of new items for all health facilities and use half of the existing items.
	Devices	DH: A default list of 16 items RRH: DH items plus 14 more (Additional file 1).	Price list (2022) –MSD	Purchase all items new for all health facilities.	Acquire half of new items for all health facilities and use half of the existing items.
Running Costs	Human Resource	Additional newborn ward personnel, organised by cadre were defined from Tanzania NEST360 HFA reports (Table 3) [23] working document on government recommended staff-to-baby ratio and refined by expert opinion.	Tanzania's health worker service scheme and national salary pay scale (2021).	Refer to Table 4.	Refer to Table 4.
		- All costed personnel were assumed to have at least 5 years of work experience. - The number of ward staff was calculated based on an 8-hour shift. - The incremental quantities accounted for maternity cover and annual leave. - Hospital management or non-newborn specific staff time was not costed.			
	Medical Supplies & Device Consumables	List of items identified from Tanzania NEST360 program data and expert opinion. Quantities were average yearly consumption estimates based on unit capacity per level of care and number of devices (Additional file 1).	Current market prices were obtained from the MSD, the UNICEF supply catalogue, and the MSH International Medical Products Price Guide.	Purchase all items for all health facilities	Acquire half new items and use half of the existing items for all health facilities
		A 10% mark-up over the economic order quantity was added.			
	Neonatal Medicines	-Tanzania National Neonatal Care guideline [24] -NEST360 Neonatal Inpatient Dataset [25]. - Expert consultation Quantities based on unit capacity, neonatal condition on admission, and medicine proportions.	Prevailing price from government procurement agency (MSD) and MSH International Medical Products Price Guide.	Purchase all items on the list for all health facilities.	Acquire half new items and use half of the existing items for all health facilities.
		Assumed a 7-day admission			
	Data & Quality Improvement system	Tanzania NEST360 alliance data and expert opinion.	Median local supplier’s unit cost.	Purchase all items on the list for all health facilities	Acquire half new items and use half of the existing items for all health facilities.
		- Data clerk will support data collection at the newborn unit. Shared resources e.g., television screen between routine data and quality improvement sessions. - Onsite mentoring will be supported by the health facility, excluded from costs. - Data storage was within the government routine data systems. e.g., DHIS-2, excluded from the costs.			
	Maintenance	Infrastructure, ward furniture and fixtures and neonatal devices.	Calculated at 3% of the set up costs.		
		Onsite maintenance by a government biomedical professional			

*Abbreviations***:** *DH* District hospital, *RRH* Regional Referral hospitals, *DHIS* District Health Information System, *MSD* Medical Stores Department, *HFA* Health Facility Assessment, *MSH* Management Sciences for Health

*Scenario B* considered the incremental setup costs of establishing 50% new SSNC units amongst the 171 hospitals (acquiring new ward furniture, fixtures, neonatal devices), while renovating the remaining 50% (and supplementing with existing resources to adhere to all government quality care standards). Running costs were based on operationalising the 50% new units while utilising existent resources (e.g., human resources, etc.) in the remaining 50% (Tables 2, 3, 4)

**Table 3:** Incremental government staffing ratios compared against baseline required for two scenarios A and B to be modelled. Baseline staffing ratios based on Health Facility Assessments conducted in seven hospitals in Tanzania by the NEST360 Alliance (*5 facilities in October 2020 and 2 facilities in February 2021*). Scenario A considered establishing and operating new additional neonatal for scale-up. Scenario B considered modifying, upgrading, or adding resources to existing neonatal units, plus setting up new additional units

**WardStaff Cadres**	Baseline	Modelling Scenarios	
		**Scenario A**	**Scenario B**
Nurse (per cots)	1:11	1:4	1:8
Nursing Assistant	1 per unit	2 per room	1 per unit
Medical Doctor/ Paediatrician	1:20 cots	1:8 cots	1:12 cots
Clinical Officer	Variable	1 per room	1 per room
Biomedical Technician	1 per hospital	1 per unit	1 (one per hospital)
Ward Clerk	Variable	2 per unit	>1 per newborn unit

Ward clerk >1 at least one health records personnel, who is not entirely attached to the newborn unit but can be shared with the health facility. Under scenario B, the ward clerk is not costed, for District Hospital it's more health system costs and for Regional Referral Hospitals one ward clerk personnel is costed.

**Table 4:** Incremental staffing requirements per hospital type, based on modelled scenarios A and B. Scenario A considered establishing and operating new additional neonatal for scale-up. Scenario B considered modifying, upgrading, or adding resources to existing neonatal units, plus setting up new additional units

**Ward Staff Cadres**	**Scenario A**		**Scenario B**	
	**District**	**Regional**	**District**	**Regional**
Nurse	13	20	7	10
Clinical Officer	4	Not charged	2	Not charged
Medical Doctor/Paediatrician	4	12	2	6
Nursing Assistant	3	9	1	5
Ward Clerk	2	2	Not charged	1
Biomedical Technician	1	1	Not charged	Not charged

#### Incremental costs: type, quantity, and unit cost data sources

Costed items and quantities were identified by reviewing Tanzanian government guidelines, neonatal inpatient data, and Health Facility Assessment data from hospitals in Tanzania implementing with NEST360, and refined by expert opinion [23, 25]. We used prevailing market prices in Tanzania, and where there were missing data, notably for some medicines and medical devices, we sourced prices from the Management Sciences for Health (MSH) international price guide and UNICEF supply catalogue. Quantities were multiplied by unit price of each item to determine total cost per cost category. Table 2 details data sources and costing scenario assumptions used to inform calculations.

#### Step 4: Return on investment

A cost–benefit ratio was used to evaluate the return on investment by directly comparing intervention costs and health benefits, both calculated in dollars at present value. This was done for scenario A only over the period 2021–2030.

For the total setup costs, infrastructure was assumed to have a useful life of 20 years, while devices and furniture to have a useful life of 5 years (Table 5). We estimated running costs assuming a gradual incremental percentage based on the number of facilities functioning from 25% in 2021 to 100% in 2030, by which time all 171 facilities would be fully functional. All costs were discounted at 3%.

We estimated the value of health benefits by monetising years of life saved. Given the modelled population was all newborns, we assumed years of life saved to equate to Tanzanian life expectancy (estimated at 66 years) [26] multiplied by number of lives saved. We compared two approaches to calculate the value of life years gained: (1) multiplied years of life saved by 2.3 times constant Gross Domestic Product (GDP) per capita for Tanzania (US$1,039 in 2021) [26–28] and (2) multiplied years of life saved by constant value of a statistical (VSL) Tanzanian life year (US$2,401) [29]. However, we used the second method for sensitivity analyses. We assumed a GDP growth of 3% annually in the 10 years (based on the last ten years) [26]. We discounted the monetary value of lives at 3% [21, 28].

##### Sensitivity analyses related to ROI

We used the Value of a Statistical Life as a measure of sensitivity analysis of the benefits once lives are monetised.

We used the LiST intervention scale up coverage from baseline to endline to 2030 to estimate the running costs as part of sensitivity analyses.

For scenario B, we ran a sensitivity analysis on projected health gains for 2025 as an outcome measure of the quality of care adhering to the two costing scenarios. We altered effectiveness of each of the priority high-impact interventions on LiST by 50% to represent scenario B, which we hypothesise is inferior in quality of care and thus effectiveness. However, we maintained effectiveness of supportive care for congenital conditions and intrapartum care because they are low-impact interventions.

#### Step 5: Financing, budget impact and implementation

We assessed the budget impact of SSNC scale-up on the cost per capita. We used the defined scale-up scenarios and a population estimate of 61.5 million [16, 26], which was compared to total health expenditure (US$40 in 2020). Cost per birth and cost per level of hospital care (i.e., District Hospital versus Regional Referral Hospitals) were all calculated as part of the unit analysis, with cost per birth estimated using the annual total births estimate of 2.15 million [16].

In addition, we explored windows of financing and presented findings to policymakers to enable targeting of the investment at national and international levels.

## Results

Results are reported according to the five-step framework.

### Step 1: Country policy imperatives and guidelines

According to Tanzania neonatal care guidelines, all District Hospitals must have a fully functional level-2 inpatient unit to care for sick and small newborns. At District level of care, the newborn unit is considered fully functional when it has a general neonatal ward (GNW), an isolation room, and a KMC room and high dependency unit (HDU). In addition to these rooms, Regional Referral Hospitals are expected to have a partial neonatal intensive care unit (NICU) and Tertiary hospitals are expected to have a full NICU for newborns requiring more intensive care.

### Step 2: Impact analysis

**Fig. 2:**
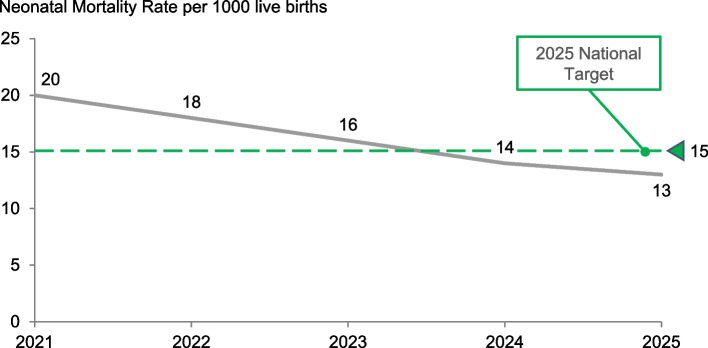
Projected lives saved with level-2 small and sick newborn care scale-up in Tanzania (including Zanzibar) using Lives Saved Tool modelling by 2025

**Fig. 3:**
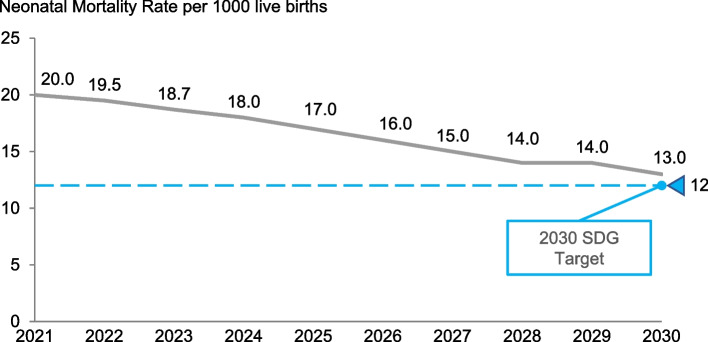
Projected lives saved with level-2 small and sick newborn care scale-up in Tanzania (including Zanzibar) using Lives Saved Tool modelling by 2030

LiST estimates showed substantial additional lives saved through scaled-up SSNC interventions. At an endline coverage of 85% with a base year of 2021 for both scenarios, there were an estimated 36,600 cumulative lives saved by 2025 (35% deaths averted) and 80,000 by 2030 (41% deaths averted). Subsequently, the NMR would reduce to 13 per 1000 live births by 2025 (Fig. 2) superseding the government target of 15. However, at the same endline coverage, the NMR remained at 13 per 1000 live births by 2030, slightly missing the SDG 3.2 target of 12 (Fig. 3). The potential additional lives saved was greatest for neonatal prematurity (48%), followed by neonatal sepsis (34%), and neonatal pneumonia (16%). Congenital conditions and intrapartum care both accounted for 3% of the lives saved.

### Step 3: Planning and costing

#### Total scale-up costs

The total one-off incremental costs for scenario A were US$166 million (US$112 million District Hospitals and US$54 million for Regional Referral Hospitals) and for scenario B were US$90 million (US$65 million and for District Hospitals and US$25 million Regional Referral Hospitals).

Once the capital assets were annualised, the total annual incremental costs for scenario A were US$57 million (US$41 million for District Hospitals and US$16 million for Regional Referral Hospitals) and for scenario B were US$28 million (US$20 million for District Hospitals and US$8 million for Regional Referral Hospitals). The cost of human resources was the main cost driver, accounting for 50% of the total costs. Set-up cost was relatively low accounting for approximately 22% of the total costs. For example, in scenario A, the equivalent annual cost of capital assets for District Hospitals was US$6 million, while the human resource cost was US$23 million.

**Fig. 4:**
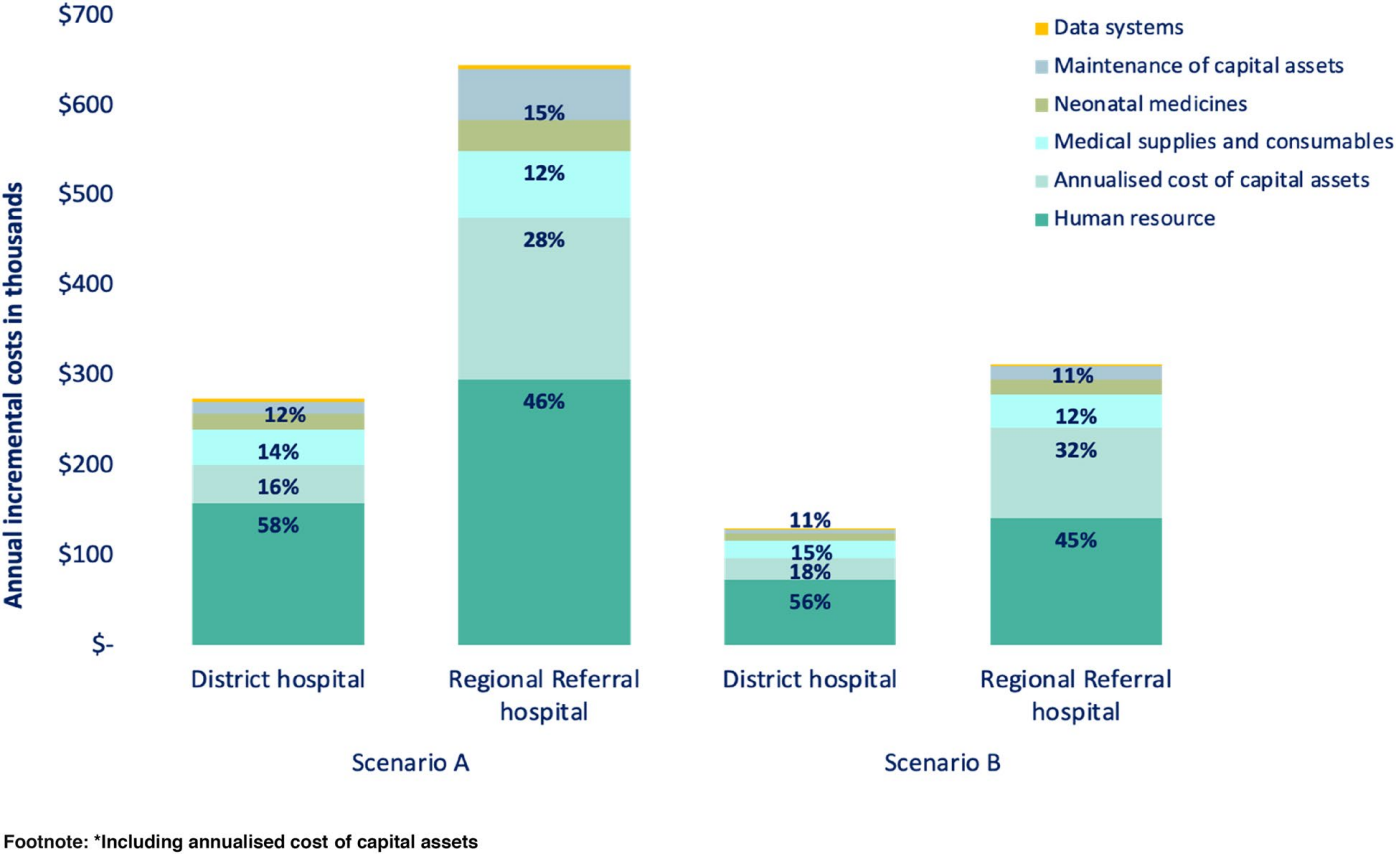
Total incremental costs* for scale-up of level-2 small and sick newborn care units in 146 District hospitals and 25 Regional Referral hospitals in Tanzania, based on two modelled scenarios and stratified by level of hospital. Scenario A considered establishing and operating new units in all 171 hospitals. Scenario B considered modifying, upgrading, or adding resources to existing neonatal units in 50% of the hospitals and establishing and operating new units in the remaining 50% of hospitals. For a district hospital the total incremental cost is US$274,000 for scenario A and US$ 130,000 for scenario B

The annualised incremental cost per hospital for scenario A were US$274,000 per District Hospital and US$645,000 per Regional Referral Hospitals and for scenario B were US$130,000 per District Hospital and US$312,000 per Regional Referral Hospitals (Fig. 4).

#### Set-up costs

**Fig. 5:**
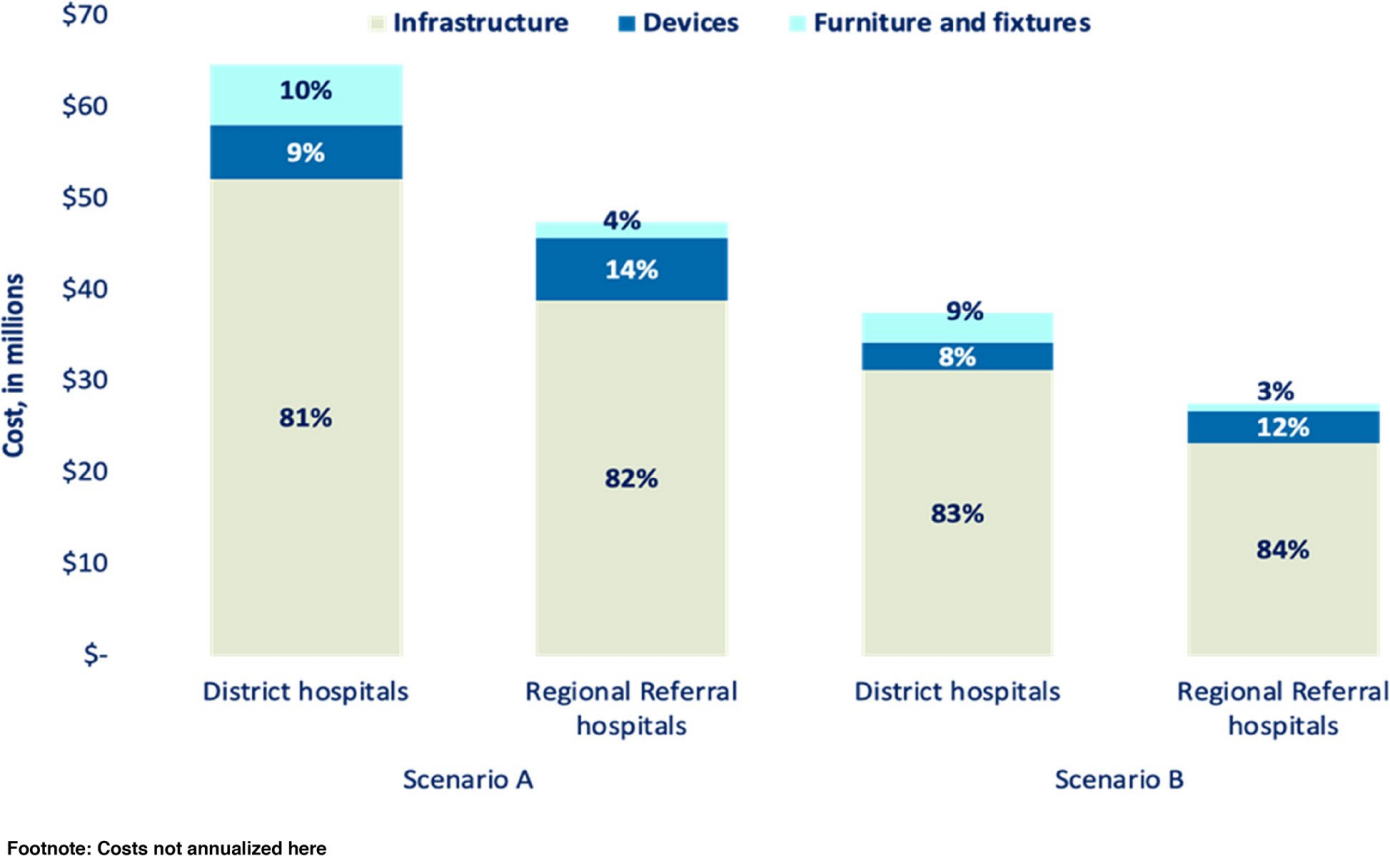
Set up Incremental costs for scale-up of level-2 small and sick newborn care units in 146 District hospitals and 25 Regional Referral hospitals in Tanzania, based on two modelled scenarios and stratified by level of hospital. Scenario A considered establishing and operating new units in all 171 hospitals. Scenario B considered modifying, upgrading, or adding resources to existing neonatal units in 50% of the hospitals and establishing and operating new units in the remaining 50% of hospitals. For a district hospital the set up incremental cost is US$448,000 for scenario A and US$ 258,000 for scenario B

At both levels of hospital and for both scenarios, infrastructure contributed to ~83% of total incremental set up costs, while neonatal devices contributed 11%. For scenario A, the total incremental cost of District Hospitals scale-up was US$65 million while for Regional Referral Hospitals was estimated to cost slightly lower at US$48 million. For scenario B the total incremental set up cost for District Hospitals was US$38 million while for Regional Referral Hospitals was US$28 million (Fig. 5).

Per hospital, the cost of either building or upgrading a District Hospitals was lower than Regional Referral Hospitals in both scenarios. For scenario A, the incremental cost per District Hospitals was US$448,000 (infrastructure; US$358,0000, ward furniture and fixtures; US$50,000, and neonatal devices; US$40,000) and US$1.9 million for Regional Referral Hospitals infrastructure; US$1.6 million, ward furniture and fixtures; US$70,000 and neonatal devices; US$ 275,000). While for scenario B costs per District Hospitals were US$258,000 and US$1.1 million (Fig. 5).

#### Running costs

Human resources were the main running cost driver at both levels of hospital and for both scenarios, accounting for approximately 60% of running costs. Medical supplies and device consumables accounted for nearly 20% of incremental running costs, and capital assets maintenance contributed to 5% of these costs. In both levels of hospital care and scenarios, neonatal medicines and data systems cumulatively contributed to 15% of the costs.

Although neonatal medicine accounted for only 8% of the total incremental cost it is important to note that antibiotics accounted for approximately 30% of this cost. In addition, the starter and refresher training for routine data collection and quality improvement accounted for 70% of the data system costs. Maintenance costs were relatively higher for Regional Referral Hospitals (15% for scenario A and 9% for scenario B), than for District Hospitals.

**Fig. 6:**
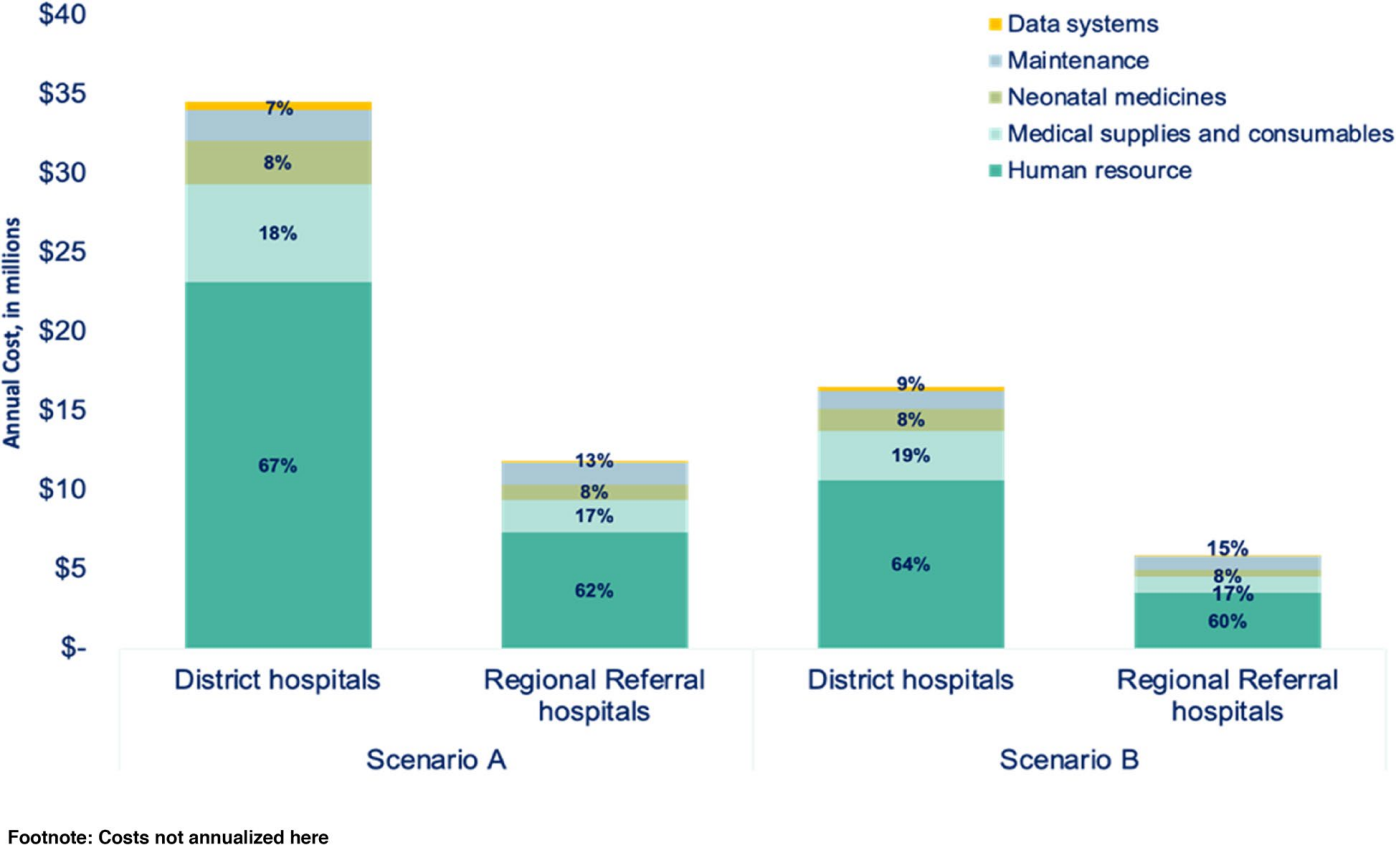
Running Incremental costs for scale-up of level-2 small and sick newborn care units in 146 District hospitals and 25 Regional Referral hospitals in Tanzania, based on two modelled scenarios and stratified by level of hospital. Scenario A considered establishing and operating new units in all 171 hospitals. Scenario B considered modifying, upgrading, or adding resources to existing neonatal units in 50% of the hospitals and establishing and operating new units in the remaining 50% of hospitals. For a district hospital the running incremental cost is US$231,000 for scenario A and US$ 106,000 for scenario B

For scenario A, total incremental annual running costs were at US$35 million for District Hospitals, and lower for Regional Referral Hospitals, at US$12 million (Fig. 6). For scenario B the incremental annual running costs were at US$17 million for District Hospitals, and lower for Regional Referral Hospitals, at US$6 million.

Incremental running cost per hospital for Regional Referral Hospital was double that of District Hospitals. For scenario A, District Hospitals was estimated at US$ 231,000 and US$ 561,000 per Regional Referral Hospital and for scenario B, operating costs were US$106,000 per District hospitals and US$ 258,000 per Regional Referral Hospital (Fig. 4).

### Step 4: Return on investment

For scenario A, the Return on Investment (i.e., CBR) was estimated between US$7–8 for every US dollar invested by 2030 assuming constant GDP per capita (no growth) (Additional file 5). The monetised benefits from lives saved, assuming each newborn would reach the estimated life expectancy in Tanzania, would be between US$12.6 billion (GDP method) in 66 years, which is a future value. However, at net present value, it would be between US$1.8 (2030). Total incremental costs to 2030 were estimated at US$376 million once set up costs are annualized based on useful life (Additional file 5). Assuming an annual GDP growth rate of 3% and similar costs the Return on Investment was estimated to be between 8 and 12 (Tables 5, 6, 7 and 8, See further details of the Return on Investment Estimation in Additional file 5)

**Table 5:** Estimating the return on investment. Estimating set up costs

**Setup Costs**	Annualized Set Up Costs (US$)	Costs To 2030 (US$)	Source
**Infrastructure**-20 years useful life	6,128,652.00	61,286,520.00	ABC COSTING
**Ward Furniture and Fixtures**-5 years useful life	1,832,054.00	18,320,540.00	ABC COSTING
**Neonatal Devices**-5 years useful life	2,770,864.00	27,708,640.00	ABC COSTING
**Total Set Up Costs**	10,731,570.00	107,315,700.00	

All set up costs were discounted at 3%

**Table 6:** Estimating running costs – including human resources, neonatal medicines, medical supplies and consumables, maintenance and data and quality improvement. We assume a gradual implementation of Scenario A as all facilities cannot be built in the first year. Total incremental Running costs per year if all is in place US$47,000,000

**Year**	**Percentage Gradual implementation of SSNC Scale up**	**Running Costs per year**
2021	25%	11,750,000.00
2022	30%	14,100,000.00
2023	35%	16,450,000.00
2024	40%	18,800,000.00
2025	55%	25,850,000.00
2026	60%	28,200,000.00
2027	70%	32,900,000.00
2028	85%	39,950,000.00
2029	90%	42,300,000.00
2030	100%	47,000,000.00
**Total Running costs**		277,300,000.00

**Table 7:** Estimating the monetised health benefits

**Inputs**	**Value**	**Approach/Source**
Lives saved by 2030	80,000	Estimated for Lives Saved Tool
Life expectancy in Tanzania	66	From World Bank indicators [26]
Years of life lost averted using Tanzania life expectancy	5,280,000.00	Lives Saved multiplied by life expectancy
Gross domestic product (GDP) per capita	US$ 1099	From World Bank Indicators
Value of a statistical life (VSL) year ) in Tanzania	US$2527.7	using GDP approach
Monetised years of life lost averted future value	US$13,346,256,000.00	GDP approach
Constant value of statistical life year in Tanzania	US$ 2401	Minimal difference with GD Papproach
Future monetised lives saved	US$12,677,280,000.00	VSL approach [29]
Present value of monetised lives	US$1,897,154,151.13	Used GDP approach as not major difference with VSL
Age to enter labour force	20	From World Bank Indicators
Productive life years when one enters labour force	46	From World Bank Indicators
Productivity gains (future value)	US$9,301,936,000.00	using GDP approach
Productivity gains (present value)	US$1,250,070,111.94	using GDP approach

*Abrreviations*: *GDP* Gross Domestic Product, *VSL* Value of a Statistical Life year

**Table 8:** Estimating the return on investment

**Social Benefits:**		
Total Benefit to 2030	US$1,897,154,151.13	Discounted At 3%
Total Cost to 2030	US$ 376,296,700	Discounted At 3%
ROI 1	5.0	
**Economic Benefits:**		
Total Benefit to 2030	US$ 1,250,070,111.94	Discounted At 3%
Total Cost to 2030	US$ 376,296,700	Discounted At 3%
ROI 2	3.3	
Total ROI	8.3	

*Abbreviations*: *ROI* Return on Investment

Using the VSL of Tanzania to monetise the lives saved we estimated the future value at US$ 14.8billion and US$2billion at net present value thus a very minimal variation between the two methods. As for the costs using the LIST to estimate running costs, the total costs were estimated at US$343 million also a very slight variation.

For scenario B, which represents reducing the effectiveness of all interventions by half, NMR by 2025 would only reduce to 17.3 per 1000 live births by both 2025 and 2030. The country would miss both the national and SDG targets if effectiveness of the interventions were lowered because of poorer quality of care (i.e., by not reaching stipulated government standards). Therefore, we did not do an ROI for this scenario.

### Step 5: Financing, budget impact and implementation

Based on total incremental costs for scenario A, we estimated the cost per birth at US$26.61 and cost per capita at US$0.93. A budget impact analysis estimated that for SSNC scale-up to 171 hospitals in Tanzania, an increase of 2% of government health expenditure per capita (from US$40.62 in 2020) would be required (United Republic of Tanzania. Ministry of Health. National Health Accounts for Financial Years 2017/18, 2018/19, and 2019/20. 2022. Government Unpulished document) [30]. Cost per death averted was estimated between US$ 4297 and US$ 5,142 by 2030 considering sensitivity analysis of the running costs.

Tanzania is negotiating for an additional US$200 million in GFF funding, earmarked for investment in RMNCH as part of their One Plan III implementation. The Tanzanian Ministry of Health and Department of Newborn and Child Health are advocating for a portion of these funds to be allocated towards the implementation of this investment case for SSNC scale-up. The COVID-19 pandemic has increased donors' interest in building resilient health systems, leading many programs in Tanzania to focus on training health workers, developing communication materials, and equipping health facilities with modern technologies.

## Discussion

More ambitious investment in the care of small and sick newborns is necessary to reach neonatal mortality targets, noting that 63 countries are currently off track for their 2030 SDG targets for neonatal survival. National scale-up of SSNC is a smart investment for Tanzania, given the high ROI demonstrated in this study of US$8–12 for every $1 invested. By reaching high coverage (85%) of high-impact small and sick newborn care, we estimated that the country would slightly miss its national SDG of reducing neonatal mortality to <12 per 1000 live births by 2030. Scaling up appears affordable at US$0.97 per capital and with a 2.3% increase in per capita health expenditure. However, delaying investment could result in ineffective implementation, quality care gaps, and failure to achieve the SDG target. There is increasing political commitment to newborn health in Tanzania, and additional funding has already been allocated by the government especially for infrastructure, but scale-up and sustaining high-quality care necessitates dependable financing.

Scaling up SSNC in Tanzania across 80% of districts was estimated to cost between US$159 million (scenario A) and US$88 million (scenario B). The annualised incremental costs per hospital for scenario A were US$274,000 per District Hospital and US$645,000 per Regional Referral Hospital. Scenario A is the 'best-case' scenario where all units were assumed to be newly built. Although this is higher set up cost, if this then lasts 20 years the investment could prevent repeated costs to renovate and rebuild every few years. Scenario B is more affordable, assuming half of existing units can be upgraded, and some units are newly built using the national neonatal floor plan. However, since many hospitals in the country are a very small room with a single nurse, and functioning below optimal levels it is plausible that this approach would not achieve the same quality and therefore lower the level of impact [23, 24].

Set up costs were largely driven by infrastructure (83% of set up costs) and running costs by human resources (60% of running costs). Infrastructure plays a critical role in SSNC outcomes, and drives set up costs, yet is necessary for quality of care – both effective service provision and respect and dignity for families and workers. Infrastructure for neonatal care has not been a focus and standard hospital floor plans, even for maternity units, may omit a newborn ward. Devices, often considered a major cost barrier to implementing quality SSNC, accounted for a surprisingly affordable cost of less than 15% of the set up costs. Maintenance systems and training are important to prevent occurrence of ‘device graveyards’ in low-resource settings. The higher device concentration in Regional Referral Hospitals may explain higher maintenance costs compared to District Hospitals, [23, 24, 31]. Some previous analyses estimated lower incremental costs for SSNC scale-up, but did not include infrastructure, furniture, and devices costs in their estimates, all critical components of SSNC [2, 27, 32, 33]. Multi-sectoral partnerships are key, particularly for infrastructure investment [31].

Human resource total incremental costs (50%) were higher than amortised infrastructure costs (22%) and this is consistent with an investment case for Primary Health Care in Kenya [21, 28], indicating the imperative for a workforce planning to implement interventions once infrastructure is in place. In Tanzania, it was estimated that 2,500 additional nurses would be required to close the scale-up gap under the recommended government policy on staff-to-newborn ratios, with the biggest gap in rural areas. Equitable human resource distribution is crucial to avoid overcrowding of urban hospitals, which can negatively impact quality of care and health outcomes [30, 34, 35]. Training of the health workforce also needs to be accounted for when planners are considering SSNC scale-up costs [32].

The return on investment for SSNC scale-up was estimated at US$8 and US$12 for every US dollar invested. This was similar to the return on investment for maternal and child health interventions (US$7) [36] and for adolescent health (US$12) [36]. Given this high ROI governments could consider allocating more funding for newborn health as part of RMNCH budgets. It is possible that set up costs for adolescent or child health interventions are less than for small and sick newborn interventions, thus seeming like cheaper investment, but the mortality gains are also lower hence the return on investments are similar. In addition, we may be likely to underestimate the benefits as we only focus on mortality and do not include morbidity averted.

We estimated a budget impact of 2.3% on top of the current government total health expenditure of US$40. This is estimate can inform budget projections for scale up of SSNC, but still subject to government actually allocating resources [33]. Adopting a phased approach to implementation of the investment case more so to reach rural and urban need, as was seen in the immunization coverage in Tanzania, could increase feasibility [30, 37]. The Tanzanian Government has already allocated new funding based on these analyses. Leveraging other support towards newborn health would also be critical. Donor funding has been a leading source of financing for health interventions in Tanzania, with priorities areas guided by the government. Donor analyses for 2002–2019 indicated US$142M support for RMNCH interventions, but newborn-specific interventions made up <US$1M of the total aid [5]. While there are efforts to track newborn funding from Official Development Assistance there are limitations as this data is self-reported from donors and may exclude other donors [5]. LMIC are not able to track details for newborn care within the National Health Accounts current set up as there are no relevant budget lines.

Tanzanian hospitals implementing a newborn health systems package with the NEST360 Alliance reported structural gaps, insufficient trained healthcare workers, and inadequate equipment at baseline [23]. Quality improvement initiatives in these hospitals have been promising [31]. Whilst focus on SSNC scale-up is high impact and returns, to reach national neonatal target the country may also need higher coverage and quality of maternity care to reach the SDG3.2 target of NMR <12. High coverage and quality of antenatal and maternity care as well as SSNC would have higher impact on neonatal mortality and also reduce stillbirths and maternal mortality [2].

Our investment case development approach had strengths, including co-developing the five-step framework with stakeholders, prioritizing interdisciplinary engagement, and utilising the comprehensive ABC costing method with updated country-specific unit costs. We also employed government-approved costed neonatal floor units to cost infrastructure. Prior studies used the LiST and One Health costing modules, which have limited cost inputs related to neonatal care [2, 27, 32, 33]. The LiST costing module includes only bag and mask and antibiotics, not the requirements for SSNC. Hence, we used LiST to estimate health benefits through lives saved but not to estimate costs while for the health benefits we estimated impact for the entire country (Mainland Tanzania plus Zanzibar), for the costing only the scale up for Mainland Tanzania was included. However, there are fewer facilities in Zanzibar and the number births in Zanzibar only account for 3% of the total births annually. Therefore, this gap is expected to have a marginal effect on the results. The Government of Zanzibar also plans to do a specific investment case.

Several challenges were encountered in the costing analyses. First, costs included in the analysis are from the provider perspective, costs incurred by the patients were not considered. Second, our focus was on level-2 care and the more substantial costs of scaling level-3 neonatal intensive units were not fully included; infrastructure and ward furniture were fully costed while neonatal device costs included only some of the devices required for level-3. Third, while we did include estimates of costs of for some health system bottlenecks such data strengthening and quality improvement, we did not attempt to cost for context specific bottlenecks such as leadership and governance.

LiST does not estimate morbidity averted nor account for disability, and while ensuring newborn survival is a global imperative, it is important for families and countries that newborns both survive and thrive. Our analyses underestimated this potential benefit. For future studies to account for morbidity averted, more cohort studies would be needed to provide reliable data on disability to be included in LiST. To ensure SSNC is incorporated into other costing exercises for health strategic plans, more comprehensive ingredient costs for SSNC inputs should be included in widely used tools such as LiST [19] and One Health [38].

Learnings from Tanzania's application of the five-step framework can guide use in other countries. Importantly Tanzania had well-defined targets and existing policies including a national neonatal unit floorplan, but lacked comprehensive neonatal guidelines for staffing ratios, medical devices, and medical drugs. A phased approach for implementation of this investment case may include focusing on rural areas at first before urban areas. It would be valuable to monitor implementation for accountability in Tanzania and to provide learnings for other countries. To track financing for newborn health, policymakers could consider including a budget line in national and potentially sub-national health budgets.

## Conclusion

Investment is crucial for countries to achieving neonatal mortality targets and reducing overall under-5 deaths with limited time to 2030, noting that SDG 3.2 for newborn survival cannot be achieved without increasing or redirecting resources to SSNC. Tanzania's success with Millenium Development Goals drives their ambition to achieve the neonatal SDG, but resources must be leveraged for SSNC implementation. Investing in neonatal health has high returns, but also moral arguments, to change outcomes for the most vulnerable citizens and reduce deaths which are affecting lose to 2.3 million families worldwide every year.

## Supplementary Information

**Additional file 1.** Neonatal floor plans. Floor plans for District hospital and regional referral hospitals costed by the Ministry of Health and Ministry of Works in Tanzania.

**Additional file 2.** Lives Saved tool interventions. List of interventions with detailed descriptions, effectiveness levels and coverages.

**Additional file 3.** List of items of health system inputs. List of items of health system inputs; ward furniture& fixtures, neonatal devices, medical supplies and device consumables, neonatal medicines and data and quality improvement systems.

**Additional file 4.** Table on Level of Care. Outlines the different level of care and what is the standard required for each level.

**Additional file 5.** a Return on investment. Step by step guide for estimation of the return on investment. b Return on investment. Step by step guide for estimation of the return on investment.

**Additional file 6.** Ethical Approval. Local ethical approval for the complex evaluation of the implementation of a small and sick newborn care package with NEST360 alliance. Table of country protocol titles and Local Ethics Committee protocol identity numbers.

## Data Availability

Not applicable.

## Abbreviations

CPAP: Continuous Positive Airway Pressure
ENAP: Every Newborn Action Plan
GFF: Global Financing Facility
LiST: Lives Saved Tool
LMIC: Low Middle-Income Countries
MSD: Medical Stores Department
NEST360: Newborn Essential Solutions and Technologies
NMR: Neonatal Mortality Rate
SDGs: Sustainable Development Goals
SSNC: Small and Sick Newborn Care
SSA: Sub-Saharan Africa
UN: United Nations
WHO: World Health Organization

## References

[1] UN Interagency Group for Child Mortality Estimation. Levels and trends in child mortality: Report 2022. New York: UN-IGME; 2023.

[2] Bhutta ZA, Das JK, Bahl R, Lawn JE, Salam RA, Paul VK, et al. Can available interventions end preventable deaths in mothers, newborn babies, and stillbirths, and at what cost? Lancet. 2014;384(9940):347–70. 24853604 10.1016/S0140-6736(14)60792-3

[3] Lawn JE, Blencowe H, Oza S, You D, Lee AC, Waiswa P, et al. Every Newborn: progress, priorities, and potential beyond survival. Lancet. 2014;384(9938):189–205. 24853593 10.1016/S0140-6736(14)60496-7

[4] World Health Organization. Every newborn: an action plan to end preventable deaths. Geneva: WHO; 2014.

[5] Kumar MB, Bath D, Binyaruka P, Novignon J, Lawn JE, Pitt C. Donor aid mentioning newborns and stillbirths, 2002–19: an analysis of levels, trends, and equity. Lancet Global Health. 2023;11(11):e1785–93. 37858588 10.1016/S2214-109X(23)00378-9 PMC10603612

[6] Lawn JE, Blencowe H, Kinney MV, Bianchi F, Graham WJ. Evidence to inform the future for maternal and newborn health. Best Pract Res Clin Obstet Gynaecol. 2016;36:169–83. 27707540 10.1016/j.bpobgyn.2016.07.004

[7] World Health Organization, United Nations Children’s Fund. Every newborn: coverage targets & milestones to 2025. Geneva: WHO; 2020.

[8] World Health Organization. Survive and thrive: transforming care for every small and sick newborn. Geneva: WHO; 2018.

[9] World Health Organization. Improving maternal and newborn health and survival and reducing stillbirth: progress report 2023. Geneva: WHO; 2023.

[10] Moxon SG, Lawn JE, Dickson KE, Simen-Kapeu A, Gupta G, Deorari A, et al. Inpatient care of small and sick newborns: a multi-country analysis of health system bottlenecks and potential solutions. BMC Pregnancy Childbirth. 2015;15:1–19. 26391335 10.1186/1471-2393-15-S2-S7 PMC4577807

[11] Global Financing Facility World Bank. Guidance Note: Investment Cases. 2016. Available from https://www.globalfinancingfacility.org/guidance-note-investment-cases. Acessed 1 Nov 2022.

[12] United Republic of Tanzania. Ministry of Health, Community Development, Gender, Elderly and Children. National Plan for Reproductive, Maternal, Newborn, Child and Adolescent Health & Nutrition (2016-2020).Tanzania-One-Plan-II. 2016. Available from https://www.globalfinancingfacility.org/sites/gff_new/files/Tanzania_One_Plan_II.pdf. Accessed 10 Oct 2022.

[13] United Republic of Tanzania. Planning Commission. The Tanzania Development Vision 2025.2000 Available on http://www.tzonline.org/pdf/theTanzaniadevelopmentvision.pdf. Accessed 10 Oct 2022.

[14] United Republic of Tanzania. Ministry of Health, Community Development, Gender, Elderly and Children. Health Sector Strategic Plan July 2021 – June 2026 (HSSP V). 2021. Available from https://mitu.or.tz/wp-content/uploads/2021/07/Tanzania-Health-Sector-Strategic-Plan-V-17-06-2021-Final-signed.pdf. Accessed 10 Oct 2022.

[15] Ogbo FA, Ezeh OK, Awosemo AO, Ifegwu IK, Tan L, Jessa E, et al. Determinants of trends in neonatal, post-neonatal, infant, child and under-five mortalities in Tanzania from 2004 to 2016. BMC Public health. 2019;19:1–12. 10.1186/s12889-019-7547-x PMC6734430 31500599

[16] United Republic of Tanzania. Ministry of Health, Community Development, Gender, Elderly and Children. National Plan for Reproductive, Maternal, Newborn, Child and Adolescent Health & Nutrition (2021/2022 - 2025/2026).Tanzania-One-Plan-III. 2021. Available from https://www.globalfinancingfacility.org/sites/gff_new/files/Tanzania-One-Plan-III.pdf. Accessed 10 Oct 2022.

[17] Fox MJ, Martorell R, Van Den Broek N, Walker N. Assumptions and methods in the lives saved tool (LiST). BioMed Central. 2011;11:1–3. 21501425 10.1186/1471-2458-11-S3-I1 PMC3231881

[18] Jo Y, Labrique AB, Lefevre AE, Mehl G, Pfaff T, Walker N, et al. Using the lives saved tool (LiST) to model mHealth impact on neonatal survival in resource-limited settings. PloS One. 2014;9(7):e102224. 25014008 10.1371/journal.pone.0102224 PMC4094557

[19] Walker N, Tam Y, Friberg IK. Overview of the lives saved tool (LiST). Springer. 2013;13:1–6. 10.1186/1471-2458-13-S3-S1 PMC3847271 24564438

[20] Mercier G, Naro G. Costing hospital surgery services: the method matters. PloS One. 2014;9(5):e97290. 24817167 10.1371/journal.pone.0097290 PMC4016301

[21] Mwai D, Hussein S, Olago A, Kimani M, Njuguna D, Njiraini R, et al. Investment case for primary health care in low- and middle-income countries: A case study of Kenya. PLoS One. 2023;18(3): e0283156. 36952482 10.1371/journal.pone.0283156 PMC10035909

[22] Tarus A, Msemo G, Kamuyu R, Shamba D, Kirby R, Palamountain K, et al. Planning and costing for furniture and devices for small and sick newborn care: systematic development of a tool. BMC Pediatr. 2023, Accepted paper in the same Supplement. 10.1186/s12887-023-04363-w PMC10652422 37968613

[23] Penzias RE, Bohne C, Gicheha E, Molyneux EM, Gathara D, Ngwala S, et al. Quantifying health facility service readiness for small and sick newborn care: comparing standards-based and WHO level-2+ scoring for 64 hospitals with NEST360 in Malawi, Kenya, Tanzania, and Nigeria*.* Abstract presented at: International Maternal & Newborn Health Conference; May 11, 2023; Cape Town, South Africa.

[24] United Republic of Tanzania. United Republic of Tanzania. Ministry of Health, Community Development, Gender, Elderly and Children. National Guideline for Neonatal Care and Establishment of Neonatal Care Unit. 2019. https://www.medbox.org/document/national-guideline-for-neonatal-care-and-establishment-of-neonatal-care-unit#. Accessed 10 October 2022.

[25] Cross JH, Bohne C, Ngwala SK, Shabani J, Wainaina J, Dosunmu O, et al. Neonatal inpatient dataset for small and sick newborn care in low- and middle-income countries: BMC Pediatr. 2023, Accepted paper in Same supplement. 10.1186/s12887-023-04341-2 PMC10652643 37968588

[26] World Bank. World Development Indicators. 2020. Available from https://databank.worldbank.org/source/world-development-indicators. Accessed 31 May 2023.

[27] Stenberg K, Sweeny K, Axelson H, Temmerman M, Sheehan P. Returns on investment in the continuum of care for reproductive, maternal, newborn, and child health. Reproductive, Maternal, Newborn, and Child Health: Disease Control Priorities, Third Edition (Volume 2). 2016. 27227218

[28] Chisholm D, Sweeny K, Sheehan P, Rasmussen B, Smit F, Cuijpers P, et al. Scaling-up treatment of depression and anxiety: a global return on investment analysis. Lancet Psychiatry. 2016;3(5):415–24. 27083119 10.1016/S2215-0366(16)30024-4

[29] Viscusi WK, Masterman CJ. Income elasticities and global values of a statistical life. J Benefit-Cost Analysis. 2017;8(2):226–50.

[30] Strasser R, Kam SM, Regalado SM. Rural health care access and policy in developing countries. Ann Rev Public Health. 2016;37:395–412. 26735432 10.1146/annurev-publhealth-032315-021507

[31] Newborn Essential Solutions & Technologies. NEST360. 2019. https://nest360.org. Accessed 20 June 2023.

[32] United Nations International Children’s Emergency Fund (UNICEF). Investing In Newborn Health in South Asia. UNICEF, New York: 2021.Available from https://www.unicef.org/rosa/reports/investing-newborn-health-south-asia. Accessed 30 May 2023.

[33] Stenberg K, Axelson H, Sheehan P, Anderson I, Gülmezoglu AM, Temmerman M, et al. Advancing social and economic development by investing in women’s and children’s health: a new Global Investment Framework. Lancet. 2014;383(9925):1333–54. 24263249 10.1016/S0140-6736(13)62231-X

[34] Norris M, Klabbers G, Pembe AB, Hanson C, Baker U, Aung K, et al. A growing disadvantage of being born in an urban area? Analysing urban–rural disparities in neonatal mortality in 21 African countries with a focus on Tanzania. BMJ Global Health. 2022;7(1):e007544. 34983787 10.1136/bmjgh-2021-007544 PMC8728407

[35] Bolan N, Cowgill KD, Walker K, Kak L, Shaver T, Moxon S, et al. Human resources for health-related challenges to ensuring quality newborn care in low-and middle-income countries: a scoping review. Global Health. 2021;9(1):160–76. 10.9745/GHSP-D-20-00362 PMC8087437 33795367

[36] Clark H, Coll-Seck AM, Banerjee A, Peterson S, Dalglish SL, Ameratunga S, et al. A future for the world’s children? A WHO–UNICEF–Lancet Commission. Lancet. 2020;395(10224):605–58. 32085821 10.1016/S0140-6736(19)32540-1

[37] Thinkwell. Immunization Costing Action Network (ICAN). The Costs of Different Vaccine Delivery Strategies to Reach Children Up to 18 Months in Rural and Urban Areas in Tanzania. Available from https://thinkwell.global/wp-content/uploads/2020/10/ICAN-Tanzania-Study-Report.pdf. Accessed 9 June 2023.

[38] Avenir Health. OneHealth Tool. Cited 2022 5/5/2022; Available from: https://www.avenirhealth.org/software-onehealth.php. Accessed 9 June 2023.

