# Supplementary material for: Investment case for small and sick newborn care in Tanzania: systematic analyses
Source: BMC Pediatr. 2023 Dec 14;23(Suppl 2):632. doi: 10.1186/s12887-023-04414-2 (PMC10722687; doi:10.1186/s12887-023-04414-2)
Supplement: Supplementary file 1 — Additional file 1. Neonatal floor plans. Floor plans for District hospital and regional referral hospitals costed by the Ministry of Health and Ministry of Works in Tanzania. [file 12887_2023_4414_MOESM1_ESM.pdf]

PRESIDENT'S OFFICE
REGIONAL ADMNISTRATION AND LOCAL GOVERNMENT

BILL OF QUANTITIES FOR THE PROPOSED CONSTRUCTION OF NEONATAL UNIT FOR COUNCIL HOSPITAL

August, 2022

GENERAL SUMMARY

GENERAL DESCRIPTIONS	AMOUNT
BILL No 01 - PRELIMINARIES ……………………..…………………	27,350,000.00
BILL No 02 - SPECIFICATIONS ……..	
BILL No 03 - MEASURED WORKS {NEONATAL UNIT} ……….	656,051,400.00
BILL No 04 - PRIME COSTS AND PROVISIONAL SUMS	18,800,000.00
**SUB-TOTAL**	**702,201,400.00**
**<u>INSURANCE CLAUSES:</u>**	
. Clause 13 - Contractor to maintain in joint names of the Employer and Contractor, Insurance Against Loss and Damages to the works by fire, earthquakes, etc.	3,000,000.00
. Clause 54 - Performance Security	2,000,000.00
SUB-TOTAL ------------------- (1)	707,201,400.00
ADD: 18% Value Added Tax (VAT) ---------------	127,296,252.00
SUB-TOTAL ------------------- (2)	834,497,652.00
**AMOUNT CARRIED TO FORM OF TENDER TShs.**	**834,497,652.00**

Signed by ………………………..…..……….. For and on behalf of …………………..……………………..……..

In the capacity of ………..………...…..……… made this ……..……… day of …..…..……… 2022

ITEM	DESCRIPTIONS OF WORKS	TSHS.
	**DESCRIPTION OF SITE:**	
A.	The site is located District/Councils WITHIN TANZANIA COUNTRY	
B.	The Contractor shall provide and maintain any necessary temporary roads; sleeper tracks; and temporary cross over during the execution of the works; clear away the same at completion and reinstate and make good any work disturbed to the satisfaction of the Local Authority and the Employer.	
C.	The Contractor shall be deemed to have visited the site and satisfied himself as to:-<br>i) The nature of the site<br>ii) The amount of bush; rubbish or debris to be cleared away before. commencement.<br>iii) The nature of proximity and size of adjoining building and property.<br>iv) The nature of existing communications by roads or otherwise.<br>v) The means of access to the site.<br>vi) The availability of land for the erection and positioning of all temporary structures; plant and materials necessary for the execution of the works.<br>vii) The source of adequate supplies of labour, plant and materials for the completion of the works.	
D.	If the Contractor wishes to execute trial holes before submitting his tender; he may do so in positions to be agreed with the Employer and at his sole expenses; including the reinstatement of the ground if so required by the Employer.	
E.	The whole of the site will be available to the Contractor immediately upon the issue of the order to commence.	
F.	Any sand; aggregate to or other building materials shall be the property of the Employer and shall not be used in the construction of the works without the written consent of the Employer.	
G.	The Contractor is to satisfy himself as to any difficulties that the site may present and to make all necessary enquiries to any point which in his opinion requires further elucidation as no claim for lack of information on any of the above will be entertained.	
	**TO COLLECTION TSHS.**	-

ITEM	DESCRIPTIONS OF WORKS	TSHS.
A.	**DESCRIPTION OF WORKS:**<br>The work within this contract comprises of: ***Substructure, Frames ,Walls,ramp,Stairs, Roof, Doors, Windows, Service Engineering, Finishings, Decorations and External Works on Construction of Neonatal Unit for Council Hospitals***	
B.	**SINGULAR AND PLURAL**<br>Word importing the singular only also includes the plural.	
C.	**LAW GOVERNING CONTRACT**<br>The contract shall be in all respect to be constructed and operated in accordance with the law of Tanzania.	
	**METHOD OF MEASUREMENT:**	
D	These Bills of Quantities have been prepared in accordance with the standard method of measurement of Building Works for East Africa first edition (metric) published by the architectural association of Kenya chapter of Quantity Surveyor Act; 1970; and applied equally to the measurement of proposed works and of variations by Quantity Surveyors.	
E	Variation of 'Builder's Work' will be subject to the same amended rates of percentage of adjustment.	
	**DEFINITIONS OF ABBREVIATIONS:**	
F	The Contractor should take due notice of the under mentioned abbreviations:-<br>mm - millimetres<br>cm - centimetres<br>M^3 - cubic meters<br>M^2 - square metres<br>M - linear metres<br>No - Number<br>Kg - Kilograms<br>P.C - Prime cost	
G	The Contractor shall allow for keeping all records appertaining to the work and shall keep on the site a daily diary recording weather conditions; temperature; visitors to the site, etc.	500,000.00
H	The Contractor is to supply to the Employer such information as he may be required in connection with the work; including statement showing the number of men employed in all trades daily; and delivery notes (stating the name of the project) for all materials delivered to the site.	800,000.00
	**TO COLLECTION TSHS.**	**1,300,000.00**

ITEM	DESCRIPTIONS OF WORKS	TSHS.
	**EMPLOYER'S INSPECTION:**	
A.	No work shall be covered up until it is inspected and approved by the Employer.	
B.	The Employer may at any time before the end of defects liability period or during any extended time where any defect are being made good, instruct the Contractor to open up; pull down; test or expose any part of the works in order to satisfy himself as to the quality of materials or workmanship used. If in the opinion of the Employer such parts are not in strict accordance with the contract documents he may order the Contractor to remove all defective work, replace with approved materials and reinstate any such part of the works and any other disturbed at his own expenses and to the entire satisfaction of the Employer. If any such parts of the works are found to be in accordance with the contract documents the Contractor will be reimbursed with the General conditions of contract.	
	**DISTURBANCE OR NUISANCE:**	
C.	The Contractor shall allow for taking all necessary precautions in the order and execution of the work so as to avoid causing disturbance or nuisance to the occupants of existing buildings and those adjacent to the works and for complying with the Employer's instructions in this respect. The Contractor shall be in tort for such nuisance and shednets.	
	**TRESPASS, DAMAGE AND CARE OF WORKS:**	
D.	The Contractor shall prevent any trespass on the opinion adjoining property and he shall take all reasonable precautions during the progress of the contract to prevent any damage to the adjoining property or plant or private roadways and to prevent material; plant; rubbish and debris; etc. collecting on the adjoining property or roadways.	2,000,000.00
E.	Should the Contractor wish to erect scaffolding or to make use of adjoining property; he shall obtain prior permission from the Employer and clear away at a completion of his work or when directed and make good any damage to his satisfaction. Except as provided for in the General conditions of contract; the Contractor; shall be held responsible for the care of works generally until their completion; including all works executed and materials deposited on the site by himself or his Sub-Contractors or supplier together with all risks arising from weather; carelessness of operatives; damages and he shall make good all such damage or loss at his own expense	
F.	The Contractor shall be responsible for the protection of any adjacent building; boundary walls; fences; services either overhead or underground and for the making good of or paying for all damage thereto; should such be caused in the course of building operations.	5,000,000.00
G.	The Contractor shall allow for making good all damage to the road; kerbs; surface water channels; etc. occasioned by heavy traffic; delivery of materials and building operations generally to the entire satisfaction of the Employer and shall be responsible for observing any by law of Local Authority regarding keeping the road free from mud; filth dirt; etc, out of the execution of the works.	-
	**TO COLLECTION TSHS.**	**7,000,000.00**

ITEM	DESCRIPTIONS OF WORKS	TSHS.
A.	**PROTECTION FROM THE WEATHER:**<br>The Contractor shall allow for covering up and protecting all new work from injury by weather or any other cause. Any damage; loss or expense caused by non-compliance with the clause shall be at sole risk of the contract.	2,500,000.00
B.	**TOOLS, PLANT AND SCAFFOLDING:**<br>Provide all necessary cranes, hoists, concrete mixer and other plant including ladder, staging, access gangways tackle, tarpaulins, tools, moulds templates and other requisites necessary for proper executing, adapting from time to time as may be necessary and maintain all plant and equipment during the course of the contract.	700,000.00
C.	The Contractor shall allow for providing adapting from time to time as may be necessary and maintaining all scaffolding scaffold boards and temporary staging, etc, necessary for the execution of the works.	300,000.00
D.	The Contractor is to provide everything necessary for the proper execution of the works according to the true intent and meaning of the drawings; etc. whether the same may or may not be particularly shown on the drawings; specifications provided that the same is reasonably to be inferred there from.	500,000.00
E.	**SITE ACCOMODATION**:<br>The Contractor shall provide and maintain any necessary temporary office accommodation required by himself and his Sub-Contractors suitably equipped with desks; chairs; drawing boards; and electric lighting and telephone.	950,000.00
F.	The Contractor shall provide and maintain for his workers latrine facilities washing and drinking water, first aid equipment's and shelters equipped with tables; benches and checking facilities all to the reasonable satisfaction of the workers and approved by the Employer and Health Authorities.	1,000,000.00
G	The Contractor shall provide and maintain any temporary storage, shed or buildings which in his opinion are necessary for himself and his Sub-Contractors for the execution of the works.	600,000.00
H	**WATER FOR THE WORKS**<br>The Contractor shall allow for all necessary clean fresh water for the works, including that required by Sub-Contractors and for any temporary plumbing metres and storage facilities and pay all charges in connection therewith and clear away on completion and make good works disturbed.	800,000.00
J	The Contractor shall allow for providing and maintaining a temporary electricity supply for the works including that required by Sub-Contractor and for any meters and fittings to give artificial lighting and power necessary for the execution of the works and pay all charges, in connection and make good all works disturbed.	800,000.00
	**TO COLLECTION TSHS.**	**8,150,000.00**

ITEM	DESCRIPTIONS OF WORKS	TSHS.
	**SITE MEETINGS**	
A	The Contractor shall attend all site meetings arranged by the Architect as per schedule. The purpose of these meetings, to be held under the administration of the Architect is to review progress and clarify any matters arising to maintain the regular progress of the works	1,200,000.00
B	At each site meeting the Contractor shall present a written report which clearly notes the progress planned and actually achieved since the last site meeting, outstanding information required and weather reports for the period since the last site meeting stating the effects, if any, on progress of the works	600,000.00
	**CO-ORDINATION MEETINS**	
C	The Contractor shall be responsible for holding co-ordination meetings with all sub-contractors engaged on this project	
D	The purpose of the co-ordination meetings, to be held under the administration of the Main Contractor, is to clarify matters etc., and to arrange wiht other trades routes of services and position of equipment and to ensure that all services are installed in the correct sequence.	600,000.00
	**TESTING:**	
E.	Allow for testing all the installations required to be tested and provide everything necessary for this purpose and leave the whole in perfect working order to the satisfaction of the Employer and Local Authority.	1,600,000.00
	**SERVIICES DRAWINGS AND TESTING**	
G	At practical completion of the works the contractor is to deposit with the Project Manager/Architect two copies of each of record drawings showing in detail as actually executed drainage, electrical, heating and all other service installations and mains, together with certificates confirming that the installations have been TESTED and conform to the specification and requirements of the statutory authorities concerned	1,000,000.00
H	The Contractor will be responsible for obtaining from his sub-contractors all relevant drawings and certificates and for obtaining and requisite additional supporting certificates from statutory authorities	
	**TO COLLECTION TSHS.**	**5,000,000.00**

ITEM	DESCRIPTIONS OF WORKS	TSHS.
A.	**WATCHING AND LIGHTING:**<br><br>The Contractor shall allow for providing and maintaining any barriesrs; hoalding; watching; lighting which must comply with the By-laws of requirements of the Local Authority and polycy regulations and the Contractor must give all requiste policies to those authorities and provide everything necessary to protect the general public workmen; plant; materials and the whole of the works	3,000,000.00
B.	No advertisement will be permitted without the written authority of the Employer.	
C.	**SIGN BOARD:**<br>The Contractor shall provide and erect a large sized sign board on the site showing the title of the contract, the name and address of the Employer; consultant, nominated suppliers and Sub-Contractor and such information as may be required by the Employer who shall provide the sign layout and colours of the Board. The board shall be repainted when necessary and removed when no longer required.	1,500,000.00
D	Allow for the associated cost for the registration of the project ;registration of all firms engaged in the projects.	4,000,000.00
E	**PROTECTION:**<br>The Contractor is required to protect works section until completion.	
F.	**REMOVING RUBBISH AND CLEANING:**<br><br>The Contractor shall make good all defects and injuries to the works, clean down external faces wash off stains to face work, clean off marks mortar and cement, clean windows inside and out, scrub floors, flush drains run and leave all parts of the works clean, free from rubbish and waste materials and perfect on completion.	300,000.00
G.	The Contractor shall clean and cart away all rubbish as it accumulate and keep the works in orderly condition to the satisfaction of the Employer	250,000.00
	**TO COLLECTION**	**9,050,000.00**
	**COLLECTION**	
	Page No. 8/1/1	-
	Page No. 8/1/2	1,300,000.00
	Page No. 8/1/3	7,000,000.00
	Page No. 8/1/4	5,000,000.00
	Page No. 8/1/5	5,000,000.00
	Page No. 8/1/6	9,050,000.00
	**BILL No. 01- PRELIMINARIES CARRIED TO GENERAL SUMMARY**	**27,350,000.00**

BILL NO: 03 - MEASURED WORKS

ITEM	DESCRIPTION	QTY	UNIT	RATE	AMOUNT
	EXCAVATION AND EARTHWORK				
A	Site clearance of small trees, shrubs and the like including grubbing up roots	1110	m²	300.00	333,000.00
	Excavating				
B	Surfaces to reduce levels average 150mm deep vegetable soil and remove from site	1110	m²	5,000.00	5,550,000.00
	Trenches in natural ground; to receive foundations; starting from reduced level				
C	Not exceeding 1.50 meters deep	242	m³	5,000.00	1,210,000.00
D	Ditto over 1.50 m not exceeding 3.0 m deep	0	m³	5,500.00	-
	Pits; to receive foundations; starting from stripped level				
E	Not exceeding 1.50 meters deep	6	m³	5,000.00	30,000.00
F	Ditto over 1.50 m not exceeding 3.0 m deep	0	m³	5,500.00	-
G	Extra over all kinds of excavations irrespective of depth for breaking up rock	1	m³	10,000.00	10,000.00
H	Backfilling; depositing and compacting in layers maximum 150mm thick impoted material	125	m³	7,000.00	875,000.00
	Surplus excavated materials				
J	Remove from the site	123	m³	7,000.00	861,000.00
	Disposal of water				
K	Keeping excavation free from all water including spring or running water		Item		100,000.00
	Plunking and Strutting				
L	Generally sides of excavation		Item		150,000.00
	Filling				
A	Sand filling in making up levels; average 150 mm thick	118	m³	10,000.00	1,180,000.00
	To Collection				**10,299,000.00**

ITEM	DESCRIPTION	QTY	UNIT	RATE	AMOUNT
	Hardcore and the like				
A.	150mm thick stone hardcore bed; leveled; compacted and sand blinded to receive polythene membrane; measured separately.	770	m²	15,000.00	11,550,000.00
	Soil Sterilization				
B.	Gladiator solution 4TC applied to hardcore bed as per manufacturer specification	770	m²	2,000.00	1,540,000.00
	Ditto; at a rate of 8 litres per linear metre 300mm width to 485x60mm deep; external backfilling of foundations	173	m	1,000.00	173,000.00
	**Concrete works**				
C.	Insitu concrete plain grade 10' mix ratio (1:4:8)<br>50mm Thick blinding	6	m²	12,000.00	72,000.00
	Plain in-situ concrete; grade 20N/sq.mm nominal mix (1:2:4)				
D.	100mm Bed	770	m²	24,000.00	18,480,000.00
E.	foundation footing	57	m³	240,000.00	13,680,000.00
	Vibrated Reinforced in-situ concrete; grade 25 nominal mix (1:11/2:3)				
F.	Column bases	2	m³	260,000.00	520,000.00
G.	Plinth beam	25	m³	260,000.00	6,500,000.00
H.	Columns	1	m³	260,000.00	260,000.00
	**REINIFORCEMENT**				
J.	BRC mesh Reference A 252; mesh 200 x 200 mm;weight 6.16 kg per square metre; 200 mm end laps in any location	770	m²	12,000.00	9,240,000.00
	Reinforcement; bars; BS 4449:1969 hot rolled round high yield steel straight or bent				
K.	16mm Diameter bars	208	kg	3,000.00	624,000.00
L.	12mm Diameter bars	1321	kg	3,000.00	3,963,000.00
M.	8mm Diameter bars	755	kg	3,000.00	2,265,000.00
	To Collection				**80,346,000.00**

ITEM	DESCRIPTION	QTY	UNIT	RATE	AMOUNT
	Formwork (Marine plywood)				
A	Vertical sides of columns;	6	m²	20,000.00	120,000.00
B	Ditto to vertical sides of beams	214	m²	20,000.00	4,280,000.00
C	Vertical sides of bed over 75mm not exceeding 150mm	157	m	20,000.00	3,140,000.00
	Walling				
	**Concrete block B.S 2028 type A; 3.5N per square millimetre; solid in cement sand** mortar (1:4)				
D	230mm Thick wall	321	m²	30,000.00	9,630,000.00
	Damp-proof Courses (DPC)				
E	230mm Wide	356	m	1,000.00	356,000.00
	Damp-proof Membrane (DPM)				
F	500Gauge polythene sheet laying on blinded hardcore with 150mm sides and end laps	770	m²	2,500.00	1,925,000.00
	**Expansion Joints:**				
G.	20mm stryropol comprehesive materials or any approved material expansion joint set vertically between walling including sealing with any approved material on both sides.	28	m	5,000.00	140,000.00
	**Sundries**				
H.	12mm Cement and sand (1:3) external rendering to concrete block wall	107	m²	7,000.00	749,000.00
J.	Prepare and apply two coats of black bituminous paint on rendered or concrete surfaces, externally	107	m²	7,000.00	749,000.00
	To Collection				**21,089,000.00**
	**COLLECTION**				
	Page 3/1/1				10,299,000.00
	Page 3/1/2				80,346,000.00
	Page 3/1/3				21,089,000.00
	**ELEMENT NO. 1 - SUBSTRUCTURE CARRIED TO SUMMARY**				**111,734,000.00**

ITEM	DESCRIPTION	QTY	UNIT	RATE	AMOUNT
	**ELEMENT NO. 2 - FRAME**				
	**Concrete work**				
	Vibrated Reinforced in-situ concrete; grade 25 nominal mix (1:1.5:3)				
A	Beams; horizontal or sloping not exceeding 15 degrees from horizontal	36	m^3	280,000.00	10,080,000.00
B	Columns	1	m^3	280,000.00	280,000.00
C.	Roofing Gutter	15	m^3	280,000	4,200,000.00
D.	Sun Shade	6	m^3	280,000	1,680,000.00
E.	150mm thick horizontal slab	12	m^2	42,000.00	504,000.00
F.	**Coping**<br>75x 300mm Coping with wire mesh 2.5mm and all [illegible]	98	m	18,500.00	1,813,000.00
	Reinforcement; bars; BS 4449:1969 hot rolled round high yield steel straight or bent				
G.	16mm Diameter bars	151	kg	3,000.00	453,000.00
H.	12mm Diameter bars	2449	kg	3,000.00	7,347,000.00
J.	10mm Diameter bars	222	kg	3,000.00	666,000.00
K.	8mm Diameter bars	1150	kg	3,000.00	3,450,000.00
	To Collection				**30,473,000.00**

ITEM	DESCRIPTION	QTY	UNIT	RATE	AMOUNT
	Sawn formwork(Marine plywood) to.				
A.	Vertical sides of column	17	m²	20,000.00	340,000.00
B.	Horizontal sides and soffites of beams	487	m²	20,000.00	9,740,000.00
C.	Horizontal soffits of suspended slab,,Gutter and Sun Shade	138	m²	20,000.00	2,760,000.00
					-
D.	Edges of slab over 75mm not exceeding 150mm high	185	m	3,000.00	555,000.00
	**Expansion Joints:**				
E.	20mm stryropol comprehesive materials or any approved material expansion joint set vertically between walling/column/slab including sealing with 3mm thick Aluminium plates or any approved material on both sides.	35	m	3,000.00	105,000.00
	To Collection				**13,500,000.00**
	**COLLECTION**				
	Page 3/2/1				30,473,000.00
	Page 3/2/2				13,500,000.00
	**ELEMENT NO. 2 - FRAME CARRIED TO SUMMARY**				**43,973,000.00**

ITEM	DESCRIPTION	QTY	UNIT	RATE	AMOUNT
	**ELEMENT NO4: WALLS**				
	**Block Work**				
	**Internal wall**				
	Concrete block B.S 2028 type A; 3.5N per square millimetre; solid in cement sand mortar (1:4)				
A.	150mm Wall	698	m²	28,000.00	19,544,000.00
	**External wall**				
	Concrete block B.S 2028 type A; 3.5N per square millimetre; solid in cement sand mortar (1:4)				
B.	230mm Wall	440	m²	30,000.00	13,200,000.00
C.	150mm thick, Parapet wall	141	m²	28,000.00	3,948,000.00
	**Window Cill**				
D.	75x250mm Window Cill with wire mesh 2.5mm and all formwork	112	m	15,500.00	1,736,000.00
	**Metal work**				
	Dry/ Glass wall Partition				
E.	10mm Thick Glass Partition complete as per Architect drawings and Details	46	m²	140,000.00	6,440,000.00
	**ELEMENT NO4: WALLS CARRIED TO SUMMARY**				**44,868,000.00**

ITEM	DESCRIPTION	QTY	UNIT	RATE	AMOUNT
	**ELEMENT NO 5: DOORS**				
	**Aluminium Glass Door**				
	Supply and fixing Alluminium door completely with its frame and accessories				
A	10mm thick overall size 1000x2800 mm high glass door	2	Nr.	420,000.00	840,000.00
	**Hardwood materials**				
B	50x150mm Frame with one labour	368	m	20,000.00	7,360,000.00
C	25 x 25mm Glazing beads	171	m	5,000.00	855,000.00
	**Solid core Flush door**				
	Doors: formica plastic laminated facing both sides cherry Mkongo or Mninga or equal aproved hardwood lipping to all edges; solid core flush door; formic; storm grey with hardwood edge strip; 45mm thick MDF doors				
D	Door size 1800x2100mm high double door	3	Nr.	604,800.00	1,814,400.00
E	Ditto size 1500x2100mm	7	Nr.	504,000.00	3,528,000.00
F	Ditto size 1000x2100mm	32	Nr.	336,000.00	10,752,000.00
G	Ditto size 800x2100mm	12	Nr.	268,800.00	3,225,600.00
	To Collection				**28,375,000.00**

ITEM	DESCRIPTION	QTY	UNIT	RATE	AMOUNT
	Ironmongery; supply and fix the following as manufactured by Newman Tonks ltd."or other equal and approved to hardwood with matching screws"				
A	150mm Aluminium butt hinges.	54	Pairs	7,500.00	405,000.00
B	150mm Aluminium door swing hinges	30	Pairs	10,000.00	300,000.00
C	3 Lever Mortice lock	40	Nr.	100,000.00	4,000,000.00
D	Two Lever Mortice lock	12	Nr.	70,000.00	840,000.00
E	Heavy duty door closer	10	Nr.	65,000.00	650,000.00
F	Alluminum push plate	12	Nr.	20,000.00	240,000.00
G	150mm long Heavy duty door stopper	40	Nr.	5,000.00	200,000.00
H	Alluminium pull plate	12	Nr.	20,000.00	240,000.00
	**Clear glass**				
J	6mm thick glass including beads glass over $0.5m^2$ not exceeding $1.00m^2$	34	m²	20,000.00	680,000.00
	To Collection				**7,555,000.00**
	**COLLECTION**				
	Page 3/5/1				28,375,000.00
	Page 3/5/2				7,555,000.00
	**ELEMENT NO 5: DOORS CARRIED TO SUMMARY**				**35,930,000.00**

ITEM	DESCRIPTION	QTY	UNIT	RATE	AMOUNT
	**ELEMENT NO 6: WINDOWS**				
	Aluminium glazing approved by the Architect; single glazed combination frame and windows, 45x50mm Aluminium section framing,all mullions and transome;epoxy power ccoat RAL 9006 finish, 6.14mm laminated glass pre assembled with stainless steel plates and screws window ironmongery,glazed beads fiber mosquito net, rubber gaskets and backer rods and fixing to mansory or concrete grounds,sealing all around with non-hardening Epdm Silicone sealant; scews bolts and fasterners				
A	Size 1800 x 1800mm high	29	Nr.	486,000.00	14,094,000.00
B	Ditto, Size 1800 x 800mm high	7	Nr.	216,000.00	1,512,000.00
C	Ditto, Size 1500 x 1800mm high	7	Nr.	486,000.00	3,402,000.00
D	Ditto, Size 1200x 1800mm high	7	Nr.	324,000.00	2,268,000.00
E	Ditto, Size 1000 x 800mm high	5	Nr.	120,000.00	600,000.00
F	Ditto, Size 800 x 800mm high	8	Nr.	96,000.00	768,000.00
	**METAL GRILL**				
	Supply and fix 32 x 32mm steel square pipe window grill painted red-oxide				
G	Size 1800 x 1800mm high	29	Nr.	226,800.00	6,577,200.00
H	Ditto, Size 1800 x 800mm high	7	Nr.	100,800.00	705,600.00
J	Ditto, Size 1500 x 1800mm high	7	Nr.	189,000.00	1,323,000.00
K	Ditto, Size 1200x 1800mm high	7	Nr.	151,200.00	1,058,400.00
L	Ditto, Size 1000 x 800mm high	5	Nr.	56,000.00	280,000.00
M	Ditto, Size 800 x 800mm high	8	Nr.	44,800.00	358,400.00
	**ELEMENT NO6:WINDOWS CARRIED TO SUMMARY**				**32,946,600.00**

ITEM	DESCRIPTION	QTY	UNIT	RATE	AMOUNT
	**ELEMENT NO.7: ROOFING**				
	**ROOF STRUCTURE**				
	Sawn soft wood; Impregnated with Preservatives				
A	50X150mm Beam	586	m	5,000.00	2,930,000.00
B	50X150mm Rafters	610	m	5,000.00	3,050,000.00
C	50X100mm Struts	662	m	4,000.00	2,648,000.00
D	50X100mm Wall Plate	110	m	4,000.00	440,000.00
E	50X75mm Purlins	1,260	m	3,500.00	4,410,000.00
	**ROOF COVERING**				
	28 gauge IT5 resincoated roofing sheets fixed to timber purlins with 150mm end laps, 1 1/2 corrugations side laps fixed with roofing nails				
F	Roof covering;sloping not exceeding 45 degrees from horizontal	880	m^2	35,000.00	30,800,000.00
	Accessories				
G.	Ridges capping	53	M	4,000	212,000
H.	Aluminium Flush 550mm girth	25	M	2,000	50,000
	**Metal works, Bolts**				
J.	16 mm diameter x 750 mm long; hooked ends nuts; washers casting into concrete	156	Nr.	5,000.00	780,000.00
K.	10 mm Thick steel plate size 200 x200mm	84	Nr.	2,000.00	168,000.00
M.	10mm diameter bolt complete with nuts and washers	10	Nr.	5,000	50,000
	To Collection				**45,538,000.00**

ITEM	DESCRIPTION	QTY	UNIT	RATE	AMOUNT
A.	Roof outlets; 100 diameter "Fulbora"	18	Nr.	30,000	540,000.00
	**WATERPROOFING TREATMENT**				
	**Cold bituminous primer; 4mm thick gammat water proofing membrane two layers opposite direction; 50mm thick polyurethane thermal insulation with fiber glass to manufactures specification protection layers with light weight concrete layed to slope sealing all cracks combs edges corners holes or the like with cold bituminous primer 150mm laps to cement and sand concrete base**				
	To falls and crossfalls or sloping not exceeding 45 degrees from horizontal				
B.	Roof slab	16	m^2	45,000	720,000.00
C.	Work around outlets not exceeding 150mm girth	10	Nr.	5,000	50,000.00
D.	To gutter; sides and bottoms	16	m^2	45,000	720,000.00
	To Collection				**2,030,000.00**
	**COLLECTION**				
	Page 3/5/1				45,538,000.00
	Page 3/5/2				2,030,000.00
	**ELEMENT NO7: ROOFING CARRIED TO SUMMARY**				**47,568,000.00**

ITEM	DESCRIPTION	QTY	UNIT	RATE	AMOUNT
	**ELEMENT NO. 08: FINISHINGS**				
	**Insitu finishings**				
	Plaster; 12mm first coat cement and sand (1:6); 3mm second coat of cement and lime (1:5) steel trowelled to smooth surfaces; internally:				
A	15mm To walls	1663	m^2	7,000	11,641,000.00
B	15mm to horizontal soffits of slab	138	m^2	7,000	966,000.00
C	15mm to sides of columns and beams	399	m^2	7,000	2,793,000.00
	External plastering cement sand (1:4) with approved plasticizer in two coats steel trowelled to smooth				
D	22mm To walls	440	m^2	7,500	3,300,000.00
E.	Ditto; for parapet walls/Gutter sides	302	m^2	7,500	2,265,000.00
	**TILES/TERRAZO/MARBLE/ EPOXY FINISHINGS**				
	Terrazo floor finish with two coats , first coat 20mm Thick in cement and sand (1:3) laid on concrete bed background,second coat with 20mm thick in cement and marble chippings (1:1.5), surface finish with carborundum stones including ebonite dividing strip 600mm centers and polishing the surface with approved chemical.				
F	40mm thick to floors level;	676	m^2	45,000	30,420,000.00
G	150mm Thick skirting	587	m	7,000	4,109,000.00
	To Collection				55,494,000.00

ITEM	DESCRIPTION	QTY	UNIT	RATE	AMOUNT
	Supply and fix Non Slippery EPOXY floor finish sheet with skirting not exceeding 150mm high complete and including clear silant, three coats of clear silent Epoxy ,base catalyst, industry Epoxy paint,TINA and all other materials as per manufacturer instructions and specification which meets drawing requirements.				
A.	40mm thick to floor	94	m²	50,000	4,700,000.00
	Glazed ceramic wall tiles with cushion edges to BS 1281 fixed to backings with cement sand mortar and pointing with white cement				
B.	400 x 250 x 8mm Tiling to walls	173	m²	30,000	5,190,000.00
	**Marble**				
C.	Supply and fixing 25mm thick Marble tiles to the concrete/hardwood worktop complete with all associated approved joints/bond materials by Project Architect	10	m²	280,000	2,800,000.00
	Gypsum plasterboard BS 1230 Pt. 2 1970 tapered wallboard self tapping galvanized drive screws				
D.	9mm Thick ceiling; horizontal; internal	770	m2	15,000	11,550,000.00
E.	Cornice	703	m	1,000	703,000.00
F.	Extra ceiling access panel	3	Nr.	5,000	15,000.00
	To Collection				24,958,000.00

ITEM	DESCRIPTION	QTY	UNIT	RATE	AMOUNT
	Sawn softwood pressure impregnated with preservatives				
A.	50x50mm brandering fixed at 600mm centre	2494	m	3,000	7,482,000.00
	**Beds and Backings**				
	Cement and sand (1:4) wood floated surface finish				
B.	12mm Backing to receive wall tiles	173	m²	4,500	778,500.00
C.	32mm Bedding to receive Epoxy floor	94	m²	4,500	423,000.00
	To Collection				8,683,500.00
	**COLLECTION**				
	Page 3/8/1				55,494,000.00
	Page 3/8/2				24,958,000.00
	Page 3/8/3				8,683,500.00
	**ELEMENT NO 08: FINISHINGS**				
	**CARRIED TO SUMMARY**				**89,135,500.00**

ITEM	DESCRIPTION	QTY	UNIT	RATE	AMOUNT
	**ELEMENT NO. 09: PAINTING AND DECORATIONS**				
	**Internal works**				
	Prepare and apply one thinned coat and two full coats of wash 'n' ware paint				
A	Plastered walls	2200	m²	8,000.00	17,600,000.00
B	Gyspum ceiling	770	m²	8,000.00	6,160,000.00
	**External works**				
	Prepare and apply one thinned coat and two full coats of weather guard paint to				
C	External wall surfaces	604	m²	7,000.00	4,228,000.00
	**Stone Cladding(Tanga stone)**				
D	Prepare and apply wall cladding stone as per drawing requirement	138	m²	50,000.00	6,900,000.00
	Varnishing; internal work; prepare and apply three coats of clear polyurethane clear varnish; wood surfaces.				
E	General surfaces	156	m²	7,000.00	1,092,000.00
F	Frames, linings and associated mouldings 200-300mm girth	539	m	7,000.00	3,773,000.00
	**ELEMENT NO. 09 : PAINTING AND DECORATIONS CARRIED TO SUMMARY**				**39,753,000.00**

ITEM	DESCRIPTIONS	UNIT	QTY	RATE	TOTAL
	**ELEMENT NO. 10: PLUMBING AND ENGINEERING INSTALLATIONS**				
	**EXTERNAL/ CORE SERVICES**				
	**Water supply connection**				
**A.**	Connection from public water supply main pipe to Elevated tank, including supply pipe, water meter, valves and other associated fittings.	Item	1	2,300,000	2,300,000
B.	Supply and install Plastic roof water Elevated tank with capacity of $10m^3$.	Nr.	1	2,000,000	2,000,000
C.	Supply and install valves, flanges, pressure ball valve and all necessary fittings for above water tank	Item	1	500,000	500,000
	**<u>Water supply Pipe</u>**				
D.	Supply and fix 50mm diameter gulvanised steel pipe, complete with fittings and supporting brackets for transfering water from water source to Eleated water tank	m	20	15,000	300,000
E.	Supply and install 50mm diameter gate valves to support the above installations	Nr.	1	55,000	55,000
	Supply and install 50mm diameter non-return valves to support the above installations	Nr.	1	60,000	60,000
F.	Supply and fix 40mm diameter gulvanised steel pipe, complete with fittings and supporting brackets for transfering water from the Eleated water tank to sanitaries including all necessary accessories.	m	35	12,000	420,000
	**TO COLLECTION**				**5,635,000**

ITEM	DESCRIPTIONS	UNIT	QTY	RATE	TOTAL
	**Internal and external service pipework**				
	Supply and fix PPR pipe (DiZAYN GROUP) and tubing class "6" to BS 4554 with screwed and socketed joints to BS 143 and 126 of approved manufacture joints to BS 143 and 126 of approved manufacture.				
	**Pipes**				
A	15mm diameter	m	34	4,000	136,000
B	20mm diameter	m	20	4,500	90,000
C.	25mm diameter	m	55	6,500	357,500
D	32mm diameter	m	58	8,000	464,000
E	40mm diameter	m	94	8,500	799,000
	**90˚ Elbow**				
F	32mm	Nr.	3	3,000	9,000
G	25mm	Nr.	8	2,500	20,000
H	20mm	Nr.	4	2,200	8,800
J	15mm	Nr.	40	2,000	80,000
	**Tee**				
K	32 x 32 x25mm	Nr.	2	3,000	6,000
L	25 x 25 x 20mm	Nr.	2	2,500	5,000
M	20 x 20 x 15mm	Nr.	5	2,200	11,000
	**Reducer**				
N	32/ 25	Nr.	2	3,000	6,000
P	25/ 20	Nr.	2	2,500	5,000
Q	20/15	Nr.	34	2,000	68,000
	Gate Valve; B.S. 1010; Part 2: wheel head; polished by manufacturer;joints to pipe; both ends screwed male iron				
R	25mm	Nr.	7	45,000	315,000
S	20mm	Nr.	5	40,000	200,000
T	15mm	Nr.	34	32,000	1,088,000
	**TO COLLECTION**				**3,668,300**

ITEM	DESCRIPTIONS	UNIT	QTY	RATE	TOTAL
A.	Supply and install 15mm diameter flexible pipe connectors to wash hand basin (WHB), water closet (WCs), etc.	Nr.	34	2,500	85,000
B.	Supply and install extra pipe fittings and accessories for the above water supply piping	Item	1	50,000	50,000
	**EXTERNAL/ CORE SERVICES**				
C.	Supply and install 100mm uPVC pipe class "B" according to BS 8301 - 1985 complete with associated fittings	m	185	15,000	2,775,000
D.	Ditto but diameter of 75mm	m	50	10,000	500,000
E	Construction of foul/waste water manholes of internal dimension 600x600mm with invert level not exceeding 1000mm comprising 200mm plain concrete grade "C" bed 230mm block wall plastered internally as per specification and drawings including heavy duty cast iron cover .	Nr.	17	200,000	3,400,000
F.	Construction of 200X200mm standard gully trap as per specification and drawings including heavy duty cast iron cover .	Nr.	15	100,000	1,500,000
G.	Construction of faul water septic tank of internal dimension 4840Lx1625Wx1800D mm, of capacity 9000Litres complete with other associated accessories	Nr.	1	3,500,000	3,500,000
H.	Construction of faul water soak away pit with internal dimension 3m Diameter and 3m Depth complete with all associated accessories	Nr.	1	3,500,000	3,500,000
	**TO COLLECTION**				**15,310,000**

ITEM	DESCRIPTIONS	UNIT	QTY	RATE	TOTAL
A.	Supply and install Galvanized steel wire ballon set on top of vent pipe and covered with mosquito gauze, 100mm diameter	Nr.	2	10,000	20,000
B.	Excavate trench not exceeding 1000 mm deep and average 500mm wide for laying sawerage pipe not exceeding 150mm and 75mm diameter including backfilling	m	235	10,000	2,350,000
	**Soil/waste water and vent pipe work**				
	Supply and install all above ground/exposed of class "B" to BS 8301 - 1985 uPVC pipe complete with associated fitting including chasing walls, pipe supports and necessary decoration for exposed pipe work				
	**Pipes;**				
	Pipes; fixing with holders to backgrounds requiring plugging				
C.	100mm diameter, uPVC pipe	m	40	15,000	600,000
D.	75mm diameter, uPVC pipe	m	14	10,000	140,000
E	50mm diameter, uPVC pipe	m	33	8,000	264,000
F.	40mm diameter, uPVC pipe	m	35	7,500	262,500
	**Elbows, 90^0**				
G.	100mm diameter, uPVC	Nr.	9	6,500	58,500
H	75mm diameter, uPVC	Nr.	10	6,500	65,000
J.	50mm diameter, uPVC	Nr.	12	5,000	60,000
K.	40mm diameter,uPVC	Nr.	18	4,000	72,000
	**Tee - Y**				
L.	100mm diameter, uPVC	Nr.	5	6,000	30,000
M	75mm diameter, uPVC	Nr.	3	5,000	15,000
N	50mm diameter, uPVC	Nr.	4	4,000	16,000
	**TO COLLECTION**				**3,953,000**

ITEM	DESCRIPTIONS	UNIT	QTY	RATE	TOTAL
	**45** Elbow				
A.	100mm diameter, uPVC	Nr.	9	6,000	54,000
B.	75mm diameter, uPVC	Nr.	10	5,000	50,000
C.	50mm diameter,uPVC	Nr.	12	4,500	54,000
D.	40mm diameter,uPVC	Nr.	18	4,000	72,000
E	Supply, install and test WC;Squatting/ asian type, rectangular body or the like with complete combination wash down outlet, dual flush cistern 4/6 litres as giberet standard, seat and cover all accessories. "S/P" trap pans; bedding uotlets in mastic; fixing with brass screws to backgrounds requiring plugging. Colour to be white. To be approved by Engineer.	Nr.	4	350,000	1,400,000
F.	Supply, install and test WC;Close coupled floor standing with complete combination wash down outlet, dual flush cistern 4/6 litres, seat and cover and all accessories. "P" or "S" trap pans; bedding uotlets in mastic; fixing with brass screws to backgrounds requiring plugging. Colour to white. To be approved by Engineer.	Nr.	4	350,000	1,400,000
G.	Supply, install, test and commission WC(disable);special care unit to meet the teste tap flow rate of 2litres/minutes of less and dual flush cistern 2.5litres or less for half flush and 3.5litres or less for full flush, including provision of testing certificates	Nr.	1	2,500,000	2,500,000
H.	Supply and install White vitreous china Hand wash basin; Countertop type - 480mm diameter Fixing with screws to backgrounds requiring plugging	Nr.	20	350,000	7,000,000
	**TO COLLECTION**				**12,530,000.00**

ITEM	DESCRIPTION	UNIT	QTY	RATE	TOTAL
A.	Shataff;ABS chrome with supreme Hose and wall holder as to RAK 32003 or the like fixing with brass screws to backgrounds plugging	Nr.	9	50,000	450,000
B.	Toilet paper holder;chrome as to RAK 21015 or the like fixing with brass screws to backgrounds plugging	Nr.	9	35,000	315,000
C.	Soap dispenser;glass wall mounted as to RAK 21033 or the like fixing with brass screws to backgrounds plugging	Nr.	9	35,000	315,000
D.	Supply and install Stainless steel Kitchen double bowl sink; 1060 x 600mm; complete with connecting accessories, fixing with screws to backgrounds requiring plugging and all necessary accessories to make good function To be approved by Enginner.	Nr.	1	300,000	300,000
E.	Supply and install Stainless steel Kitchen Single bowl sink; 1000 x 600mm; complete with connecting accessories, fixing with screws to backgrounds requiring plugging and all necessary accessories to make good function. To be approved by Enginner.	Nr.	1	250,000	250,000
F.	Supply install, test and commissioningl utensile cleaning sink (ceramic body)with double bowl 1050 x 600mm, complete with Faucet, drain port and all other necessary accessories and fittings.	Nr.	1	450,000	450,000
	TO COLLECTION				2,080,000

ITEM	DESCRIPTION	UNIT	QTY	RATE	TOTAL
A	Supply and install a shower mixer set comprising: 1.5m plastic flexible hose pipe, hand/wall mounted shower spray, sliding bar and soap holder complete with all necessary accessories.	Set	7	220,000	1,540,000.00
	TO COLLECTION				1,540,000
	**COLLECTIONS**				
	Page 3/10/1				5,635,000
	Page 3/10/2				3,668,300
	Page 3/10/3				15,310,000
	Page 3/10/4				3,953,000
	Page 3/10/5				12,530,000
	Page 3/10/6				2,080,000
	Page 3/10/7				1,540,000
	**ELEMENT NO. 10 : PLUMBING AND ENGINEERING INSTALLATIONS CARRIED TO SUMMARY**				**44,716,300.00**

ITEM	DESCRIPTION	QTY	UNIT	RATE	AMOUNT (TZS)
	**ELEMENT NO. 11: ELECTRICAL INSTALLATIONS**				
	**Main Distribution**				
	**Supply and install the followings:**				
A.	Isolator switch 200A rated and specifications as ABB/SCHENEIDER/HAGER or approved equivalent Shown as MSB	Nr.	1	350,000	350,000
	**Main Cabling**				
	Supply and install main cables; 4 core 50mm² PVC/SWA/PVC/Cu cables complete with glands, lugs and shrouds and all other accessories :				
B	From Main switch to ISOLATOR	m	50	140,000	7,000,000
	Supply and install main cables; 4 core 25mm² PVC/SWA/PVC/Cu cables complete with glands, lugs and shrouds and all other accessories :				
C	From ISOLATOR to Distribution board	m	60	140,000	8,400,000
	**Distribution boards**				
D.	4way TPN distribution board with MCB outgoers and 100/300mA TPN RCCB incomer as MEM and as per schematics and specifications	Nr.	3	630,000	1,890,000
	**Earthing**				
E.	Provide adeguate earthing to the entire electrical installations to each individual Distribution board as per current IEEE regulations and to conform with TANESCO requirements.this must include construction of manhole for earthing improvement	item	3	100,000	300,000
	**TO COLLECTION**				**17,940,000**

ITEM	DESCRIPTION	QTY	UNIT	RATE	AMOUNT (TZS)
A.	Lighting outlet point - including Exit Signs	Nr.	47	10,000	470,000
B.	Switched socket outlet point	Nr.	61	10,000	610,000
C.	DP outlet point	Nr.	7	25,000	175,000
	**LIGHT FITTING**				
D.	Electrical fan as Panasonic made complete with regulator and hook or equal and approved	Nr.	15	170,000	2,550,000
E.	18W LED surface mounting round down light as Opple made	Nr.	58	30,000	1,740,000
F.	LED Bulkhead -E20W -6500-FR-GP-WH as Opple made or equal and approved	Nr.	18	55,000	990,000
G.	LED Tube light 2x18W complete with its fitting as Opple made or equal	Nr.	28	40,000	1,120,000
H.	LED Tube light 1x18W complete with its fitting as Opple made or equal	Nr.	22	25,000	550,000
	**SWITCHES AND OUTLETS**				
	**Supply and Install the following Switch and Sockets as HAGER, ABB, MEM EATON & MK**				
J.	10A 1gang 1way switch	Nr.	12	5,000	60,000
K.	10A 2gang 1way switch	Nr.	20	6,000	120,000
L.	10A 2gang 2way switch	Nr.	2	6,000	12,000
M.	10A 3gang 1way switch	Nr.	3	6,000	18,000
N.	20A DP switch with neon indicator for AC	Nr.	10	15,000	150,000
P.	13A twin switched socket outlet	Nr.	46	15,000	690,000
	**TO COLLECTION**				**8,000,000**

ITEM	DESCRIPTION	QTY	UNIT	RATE	AMOUNT (TZS)
	**WIRE**				
	Supply and Installation Cables for 1.5sqmm 2.5sqmm and 4sqmm should be EURO or other equal approved				
A	Single core wire 1.5sqmm - Red	1300	m	1,000	1,300,000
B	Single core wire 1.5sqmm - Black	1300	m	1,000	1,300,000
C	Single core wire 1.5sqmm -green	1300	m	1,000	1,300,000
D	Single core wire 2.5sqmm - red	800	m	1,200	960,000
E	Single core wire 2.5sqmm	800	m	1,200	960,000
F	Single core wire 2.5sqmm green	800	m	1,200	960,000
	**TO COLLECTION**			**sub total**	**6,780,000**
	**COLLECTION**				
	Page 3/11/1				17,940,000
	Page 3/11/2				8,000,000
	Page 3/11/3				6,780,000
	**ELEMENT NO. 11 : ELECTRICAL INSTALLATIONS CARRIED TO SUMMARY**				**44,980,000.00**

ITEM	DESCRIPTIONS	UNIT	QTY	RATE	TOTAL
	**ELEMENT NR. 12: AIR CONDITIONING INSTALLATIONS**				
	**Single Split Air Conditioners with Wall Mounted type Indoor/ Fan Coil Unit: (HEATING AND COOLING)**				
	Supply, install, test and commission Single Split Air conditioner Each Set shall be completed with outdoor air-cooled condensing unit, indoor Wall Mounted type fan coil unit and wired controller/ [illegible] remote controller, AVS Voltage Surge protector including all supports and associated fittings and accessories to be approved by Engineer.				
A.	Cooling Capacity: 2.8kW (9000Btu/h)	Nr.	4	1,450,000	5,800,000
B.	Cooling Capacity: 4.5kW (15000Btu/h)	Nr.	1	1,800,000	1,800,000
C.	Cooling Capacity: 7.1kW (24000Btu/h)	Nr.	5	2,100,000	10,500,000
	**Refrigerant Pipes and Fittings to BS 2871**				
	Supply and install refrigerant copper pipes complete with all associated fittings like elbows etc and all supports, including amaflex insulation 25mm thick.				
D.	12.7mm diameter	m	28	18,000	504,000
E	9.5mm diameter	m	30	16,000	480,000
F	6.5 mm diameter	m	58	15,000	870,000
	**Condensate Drainage Pipes(PVC)**				
	Supply and install condensate drainage PVC pipes complete with associated fittings i.e. tee, elbows reducers etc so as to make the system work perfectly				
G	20mm diameter PVC pipe	m	65	3,000	195,000
	**TO COLLECTION**				**20,149,000**

ITEM	DESCRIPTIONS	UNIT	QTY	RATE	TOTAL
	**Trunking**				
A.	Supply and install 120 x 69mm galvanized steel trunking for accommodating external insulated pipe work for outdoor A/C system including corners, joints, wall plates, pipe holders and all necessary fittings.	m	35	2,000	70,000
	**Tray**				
B.	Supply and install 300mm wide, 25mm height and 2.5mm thick, galvanised steel, medium duty perforated cable tray with necessary supporting frame for supporting refrigerant pipes (Horizontal runs) between indoor and outdoor units, complete with all accessories including ties, bolts, nuts and supporting frames on walls/Concrete floor.	m	65	30,000	1,950,000
C.	Supply and install the mounting brackets, supports and other fittings for outdoor units	Nr.	10	30,000	300,000
	**Wiring**				
D.	Supply and install control and power cables between units and DP switch located near the door position.	m	50	40,000	2,000,000
E.	Supply and install control and power cables between indoor units and outdoor units located.	m	65	40,000	2,600,000
F.	Supply and install of Heat recovery ventilator, Ceiling mounted Duct type, with automatic changeover function (Heat exchange/Bypass) and all accessories temperature exchange efficiency 75%, Air flow rate 500m3/hr at 100Pa ESP.	Nr.	2	5,000,000	10,000,000
	**TO COLLECTION**				**16,920,000**

ITEM	DESCRIPTIONS	UNIT	QTY	RATE	TOTAL
**A.**	Supply and install of Heat recovery ventilator, Ceiling mounted Duct type, with automatic changeover function (Heat exchange/Bypass) and all accessories temperature exchange efficiency 75%, Air flow rate 800m3/hr at 100Pa ESP.	Nr.	2	8,000,000	16,000,000
	Supply and install ventilation duct in galvanised steel plate, with flanged joints air-tightened with approved long lasting sealing compound in accordance with BS requirements, including straps, rawl bolts, screws and brackets for suspension. Plate thickness 0.8mm and flexible duct				
B.	Round duct 250mm diameter	m	10.0	40,000	400,000
C.	Round duct 280mm diameter	m	10.0	40,000	400,000
D.	Supply and install external air grille 250x250mm, maximum noise 45 db	Nr.	4.0	150,000	600,000
E.	Supply and install 400x400mm square air diffuser	Nr.	4.0	200,000	800,000
F.	Supply and install 200mm diameter disc valve	Nr.	4.0	200,000	800,000
	**TO COLLECTION**				**19,000,000**
	**COLLECTION**				
	Page 3/12/1				20,149,000
	Page 3/12/2				16,920,000
	Page 3/12/3				19,000,000
	**TOTAL FOR ELEMENT NR. 12: HEATING, COOLING AND VENTILATION INSTALLATION**				**56,069,000**

ITEM	DESCRIPTION	QTY	UNIT	RATE	AMOUNT (TZS)
	**ELEMENT NR. 13: LOCAL AREA NETWORK**				
	**Supply and installation of the following Equipments as per drawings**				
A.	Dual RJ 45 wall face plate complete with modules, spring shutter and cabling field for Cat 6 cabling	12	Nr.	25,500	306,000
B	Single backbox streel mounting boxes 35mm	12	Nr.	1,500	18,000
C	4-pair unshielded twisted pair cable(UTP), Cat6 (305M)	495	m	2,000	990,000
D	24 ports Rj45 data and voice patch panel	1	Nr.	200,000	200,000
E.	1m RJ45 patch code	24	Nr.	8,000	192,000
F.	3m RJ45 patch code	24	Nr.	12,000	288,000
G.	Patch guide (cable organizer)	1	Nr.	32,500	32,500
H.	9U voice and data cabinet with one (1) heat extract fans and at least 4 ways power distribution unit (PDU) and glass door 9U 19'' (480X600X440)mm	1	item	600,000	600,000
J	Allow PVC conduits pipe 3/4'' installation for Data	80	Nr.	1,000	80,000
K	Labeling Machine and Tape	1	Nr.	95,000	95,000
L	Cable tie	1	pkt	25,000	25,000
M	Provide adequate earthing to voice cabinet as per IEE Regulations (Two Eathrod (16mm) and Eathware (4mm))	1	Lot	200,000	200,000
	**Configuration, test and commission:**				
N.	WS-C2960X-24PS-L catalyst 2960-x switch,cisco switch catalyst2960-X48 Gig EPoE 370w, 4x1G SFP LAN Based.	1	Nr.	4,900,000	4,900,000
P	1000 VA (1 kVA) / 900 Watt (0.9 kW) - UPS and Backup batteries	1	Nr.	1,500,000	1,500,000
	**TOTAL FOR ELEMENT NR. 13: LOCAL AREA NETWORK INSTALLATION**				**5,100,000**

ITEM	DESCRIPTION	QTY	UNIT	RATE	AMOUNT (TZS)
	**ELEMENT NR. 14: NURSE CALL SYSTEM INTALLATIONS**				
	**PRELIMINARIES**				
A	Allow for site survey and installation	1	Item	3,700,000	3,700,000
	**Supply, install, test and commission the following:**				
B	Display for Nurse calling Center	2	Nr.	756,000	1,512,000
C	Nurse Call Control Unit	6	Nr.	300,000	1,800,000
D	Bedside call unit with 3metre flexible tails	19	Nr.	160,000	3,040,000
E	Nurse call overdoor lights	2	Nr.	110,000	220,000
F	Power supply with 8 ports	1	Nr.	650,000	650,000
G	Bedhead trunking Unit	58	Nr.	380,000	22,040,000
H	Cabling and associated accessories for the complete [illegible]	450	Nr.	3,000	1,350,000
	**Installation works**				
J	Allow for site survey and installation	1	Item	9,700,000	9,700,000
	**TOTAL FOR ELEMENT NR. 14: NURSE CALL SYSTEM INSTALLATION**				**44,012,000**

ITEM	DESCRIPTIONS	UNIT	QTY	RATE	TOTAL
	**ELEMENT NR. 15: GAS SYSTEM INTALLATIONS**				
	**Wall Mounted Terminal Units**				
	Supply, install and test of Wall Mounted Terminal Units for the gases installation				
A.	Oxygen	Nr.	4	190,000	760,000
B.	Medical Air	Nr.	4	200,000	800,000
C.	Vacuum	Nr.	4	190,000	760,000
	**Locable Line Valves as per specification**				
	Lockable Line Valves				
	Supply, install and test of Locable line Valves for the gases installation				
D.	15MM	Nr.	1	220,000	220,000
E.	22MM	Nr.	1	332,000	332,000
F.	35MM	Nr.	1	664,000	664,000
	**PIPING WORK**				
	Supply, install and test Copper piping of different sizes for distribution and supply of medical gas to various areas in the hospital. The Copper pipes shall be solid drawn, seamless, deoxidised, nonarsenical, half hard, tempered and degreased material conforming to BS-6017/1981 and manufactured as per BS-2871/1971 The Copper pipe shall be supplied with Lloyd's test certificate and installed as per HTM -02-01 specifications with utmost clenliness. Color codification as per BS 1710. The piping shall be complete with all fittings i.e. elbow, tee, reducer, socket, union, flange and insulation where necessary etc conforming to HTM 02-01				
	Copper pipes				
G.	D15	m	64	30,000	1,920,000
H.	D22	m	62	55,000	3,410,000
J.	D35	m	64	100,000	6,400,000
	**TOTAL FOR ELEMENT NR. 15: GAS SYSTEM INSTALLATION**				**15,266,000**

ITEM	DESCRIPTION	PAGE	AMOUNT
	**MEASURED WORKS SUMMARY**		
	ELEMENT NO. 01 - SUBSTRUCTURE	3/1/3	111,734,000.00
	ELEMENT NO. 02 - FRAME	3/2/1.	43,973,000.00
	ELEMENT NO. 04 - WALLS	3/4/1.	44,868,000.00
	ELEMENT NO. 05 - DOORS	3/5/1	35,930,000.00
	ELEMENT NO. 06 - WINDOWS	3/6/1	32,946,600.00
	ELEMENT NO. 07 - ROOF	3/7/1.	47,568,000.00
	ELEMENT NO. 08 - FINISHINGS	3/8/2.	89,135,500.00
	ELEMENT NO. 09 - PAINTING AND DECORATIONS	3/9/1.	39,753,000.00
	**SERVICE WORKS**		
	ELEMENT NO. 10 - PLUMBING INSTALLATION		44,716,300.00
	ELEMENT NO. 11 - ELECTRICAL INSTALLATION		44,980,000.00
	ELEMENT NO. 12 - A.C INSTALLATION		56,069,000.00
	ELEMENT NO. 13- LAN INSTALLATION		5,100,000.00
	ELEMENT NO. 14 - NURSE CALL SYSTEM INSTALLATION		44,012,000.00
	ELEMENT NO. 15 - GAS SYSTEM INSTALLATION		15,266,000.00
	**BILL NO.3 - MEASURED WORKS CARRIED GENERAL SUMMARY**	**T.Shs**	**656,051,400.00**

TEM	DESCRIPTION	QTY	UNIT	RATE	AMOUNT
	**BILL NR.4: PRIME COST AND PROVISIONAL SUM**				
	The following Prime Cost Sums are for the works to be carried out by Statutory Authorities				
A	Electrical Connection and provision of meter		Sum		2,000,000.00
B	**Add:** for profit	10	%		200,000.00
C	**Add:** for general attendance		Item		100,000.00
	The following Provisional Sums are for the works or costs which cannot entirely be foresees, defined or detailed during the preparation of Bills of Quantities and should be used in whole or in part at the discretion of the Architect:				
D	Allow sum for LOGO (NEONATAL UNIT)		Sum		3,000,000
E	Progress Photographs		Sum		1,500,000
F	Testing of Materials.		Sum		2,000,000
G	Allow for a contingency to be expended or deducted as instructed by the Architect		Sum		10,000,000
	**BILL NR.4- PC AND PROVISIONAL SUMS WORK CARRIED TO GENERAL SUMMARY**				**18,800,000.00**
