# Supplementary material for: Investment case for small and sick newborn care in Tanzania: systematic analyses
Source: BMC Pediatr. 2023 Dec 14;23(Suppl 2):632. doi: 10.1186/s12887-023-04414-2 (PMC10722687; doi:10.1186/s12887-023-04414-2)
Supplement: Supplementary file 2 — Additional file 2. Lives Saved tool interventions. List of interventions with detailed descriptions, effectiveness levels and coverages. [file 12887_2023_4414_MOESM2_ESM.docx]

**Table 1: Interventions for impact modelling within the Lives Saved tool for Small and Sick Newborns Care.**

| **Intervention** | **Baseline Coverage (2021) %** | **Target Coverage (2025) (2030) %** | **Intervention Effectiveness** | **Health Impacts Assessed** |
| --- | --- | --- | --- | --- |
| **Impact on Death due to Prematurity** | | | | |
| Kangaroo Mother Care | 10 | 10 | 51% (Note: 47% affected fraction) | Increased percent of neonates receiving proper Intensive continuous skin-to-skin contact between a mother and her newborn as well as frequent and exclusive breastfeeding. |
| Care for prematurity (now called small and sick newborn care) (including CPAP/respiratory) | 10 | 85 | 90% | Increased (all) percent of neonates who have access to and receive hospital-based full supportive care, including feeding support/IV fluids, infection prevention/management, oxygen provision, management of neonatal jaundice, nasal CPAP/IPPV (as required), and surfactant for respiratory distress syndrome |
| **Impact on Death due to Neonatal Sepsis** | | | | |
| Injectable antibiotics for neonatal sepsis | 10 | 10 | 90% | Increased percent of neonates with suspected sepsis/pneumonia treated with injectable antibiotics. |
| Supportive care for neonatal sepsis | 10 | 85 | 90% | Increased percent of neonates with suspected sepsis/pneumonia treated with hospital-based full supportive care, including oxygen, IV fluids, IV antibiotics, blood transfusion, phototherapy as needed. |
| **Impact on Death due to Intrapartum Care** | | | | |
| Supportive care for intrapartum care | 10 | 85 | 10% | Care for birth asphyxia through surgery |
| **Impact on Death due to Congenital Conditions** | | | | |
| Full supportive care for congenital conditions | 10 | 85 | 10% | Care through surgery |
