# Supplementary material for: Investment case for small and sick newborn care in Tanzania: systematic analyses
Source: BMC Pediatr. 2023 Dec 14;23(Suppl 2):632. doi: 10.1186/s12887-023-04414-2 (PMC10722687; doi:10.1186/s12887-023-04414-2)
Supplement: Supplementary file 4 — Additional file 4. Table on Level of Care. Outlines the different level of care and what is the standard required for each level. [file 12887_2023_4414_MOESM4_ESM.docx]

**Table 1:** World Health Organization definition of levels of newborn care and interventions

| **Level of newborn care** | **Scope of care** |
| --- | --- |
| Level 1: Essential newborn care | Services include immediate care at birth; thorough drying, skin-to-skin contact, delayed cord clamping; resuscitation when needed; early initiation and support for exclusive breastfeeding; routine care (Vitamin K, eye care, vaccinations, weighing, clinical examinations); prevention of mother-to-child transmission of HIV; assessment, management and referral of bacterial infections, jaundice and diarrhoea, feeding problems, birth defects and other problems; pre-discharge advice on mother and baby care and follow-up. |
| Level 2: Special inpatient newborn care | Services include: thermal care; comfort and pain management; kangaroo mother care (<2500 g irrespective of stability); assisted feeding; safe administration of oxygen; prevention of apnoea; detection and management of neonatal infection, hypoglycaemia, jaundice, anaemia and neonatal encephalopathy; seizure management; safe administration of intravenous fluids; detection and referral management of birth defects.; |
| + Transition to intensive care | Continuous positive airway pressure; exchange transfusion; detection and management of necrotizing enterocolitis; specialized follow-up of infants at high risk (including preterm infants). |
| Level 3: Intensive critical newborn care | Services include: advanced feeding support; mechanical/assisted ventilation, including intubation; screening and treatment for retinopathy of prematurity; surfactant treatment; investigation and management of birth defects; paediatric surgery; genetic services. |

**Abbreviations**: WHO: World Health Organization; HIV: Human Immuno-deficiency Virus

Reference: World Health Organization. Born Too Soon: Decade of Action on Preterm Birth [Internet]. 2023 https://www.who.int/publications/i/item/9789240073890.
