# Supplementary material for: Investment case for small and sick newborn care in Tanzania: systematic analyses
Source: BMC Pediatr. 2023 Dec 14;23(Suppl 2):632. doi: 10.1186/s12887-023-04414-2 (PMC10722687; doi:10.1186/s12887-023-04414-2)
Supplement: Supplementary file 5 — Additional file 5. a Return on investment. Step by step guide for estimation of the return on investment. b Return on investment. Step by step guide for estimation of the return on investment. [file 12887_2023_4414_MOESM5_ESM.docx]

**RETURN ON INVESTMENT COSTS AND BENEFIT INPUTS.**

**A Cost inputs for both 146 district hospital and 25 regional referral hospitals**

| **Set Up Costs** | **Annualized Set Up Costs in USD** | **Costs to 2030 in USD** | **Source** |
| --- | --- | --- | --- |
| Infrastructure-20 years useful life | 6,128,652.00 | 61,286,520.00 | ABC costing method |
| Ward Furniture and Fixtures-5 years useful life | 1,832,054.00 | 18,320,540.00 | ABC costing method |
| Neonatal Devices-5 years useful life | 2,770,864.00 | 27,708,640.00 | ABC costing method |
| TOTAL SET UP COSTS | 10,731,570.00 | 107,315,700.00 |  |
| Total Running costs | 47,000,000 (if all facilities are built but this is estimated gradually | 268,981,000 |  |
| Total cost |  | 376,296,000 |  |

All set up costs and running costs were discounted at 3%.

Total incremental Running costs per year if all is in place, $47,000,000 but percentages estimation assumptions were used to gradual estimate the incremental costs of the running costs to 2030 for Scenario A as all facilities cannot be built in the first year.

**B. Benefits**

| **Input** | **Value** | **Source** | **Notes** |
| --- | --- | --- | --- |
| Lives Saved by 2030 | 80,000 | Lives Saved tool output |  |
| Life Expectancy in Tanzania | 66 | World Development Indicators 2021 -Tanzania |  |
| YLL Averted using Tanzania Life Exp | 5,280,000.00 |  |  |
| Monetizing YLL Averted | | | |
| GDP Per Capita | 1099 | World Development Indicators 2021 -Tanzania |  |
| Value of a statistical life year (USD) in Tanzania using GDP approach | 2527.7 | 2.3. times GDP per capita |  |
| Monetized YLL Averted Future value | $13,346,256,000.00 |  | GDP approach |
| Constant Value of Statistical life year in Tanzania | 2401 | Viscusi W. K. and Masterman C. J., “Income elasticities and global values of a statistical life,” J. Benefit-Cost Anal., vol. 8, no. 2, pp. 226–250, 2017. | Minimal difference with GDP approach-so we used GDP approach. |
| Present Value of monetized lives | $1,897,154,151.13 | (3% discount rate -BMGF reference case) | Used GDP approach as not major difference with VSL |
| Productivity Benefits | | | |
| Age to enter labour force | 20 | World Development Indicators 2021 -Tanzania |  |
| Productive life years when one enters labour force | 46 |  |  |
| Productivity gains (future value) | $9,301,936,000.00 |  | using GDP approach |
| Productivity gains (present value) | $1,250,070,111.94 |  | using GDP approach |

Scenario A in consideration- establishing and operating new additional neonatal for scale-up

**C. Return on Investment (ROI)**

| Social benefits |  |  |
| --- | --- | --- |
| TOTAL BENEFIT to 2030 | $1,897,154,151.13 | Discounted at 3% |
| TOTAL COST to 2030 | $ 376,296,700 | Discounted at 3% |
| ROI 1 | 5.0 |  |
| Economic benefits |  |  |
| TOTAL BENEFIT to 2030 | $1,250,070,111.94 | Discounted at 3% |
| TOTAL COST to 2030 | $ 376,296,700 | Discounted at 3% |
| ROI 2 | 3.3 |  |
| Total ROI | 8.3 |  |

**D. Sensitivity Analyses**

| **Input** | **Value** | **Source** | **Assumptions** |
| --- | --- | --- | --- |
| **Monetizing YLL Averted** | | | |
| GDP Per Capita | 1428.9 | World Development Indicators 2021 -Tanzania | Assumed a 3% growth in GDP annually |
| Value of a statistical life year (USD) in Tanzania using GDP approach | 3286.7 | 2.3. times GDP per capita |  |
| Monetized YLL Averted Future value | $17,352,561,600.00 |  | GDP approach |
| Present Value of monetized lives | $2,466,645,647.46 | (3% discount rate -BMGF reference case) | Used GDP approach as not major difference with VSL |
| PRODUCTIVITY BENEFITS | | | |
| Productivity gains (future value) | $ 12,094,209,600 |  | using GDP approach |
| Productivity gains (present value) | $ 2,261,917,045.66 |  | using GDP approach |

**E. Return on Investment (ROI) with Sensitivity Analyses**

| **Social Benefits** |  |  |
| --- | --- | --- |
| TOTAL BENEFIT to 2030 | $2,466,645,647.46 | Discounted at 3% |
| TOTAL COST to 2030 | $ 376,296,700 | Discounted at 3% |
| ROI 1 | 6 |  |
| TOTAL BENEFIT to 2030 | $ 2,261,917,045.66 | Discounted at 3% |
| TOTAL COST to 2030 | $ 376,296,700 | Discounted at 3% |
| ROI 2 | 6 |  |
| Total ROI | 12 |  |

**Return On Investment Output**

*RETURN ON INVESTMENT USING GDP APPROACH TO ESTIMATE THE VSL*

*FOR EVERY DOLLAR INVESTED, TANZANIA GAINS $8 BACK ASSUMING NO GDP GROWTH*

*ASSUMING A 3% ANNUAL GDP GROWTH TO 2030, ROI IS ESTIMATED AT 12 FOR EVERY DOLLAR INVESTED*
